# Bioactive preservative nano-packaging films based on food wastes of orange peels and Shrimp for apple (*Malus domestica* var. Anna) fruit quality and storage

**DOI:** 10.1186/s40643-025-00890-9

**Published:** 2025-06-06

**Authors:** Mohamed S. Hasanin, Mahmoud Emam, M. A. Ahmed, F. M. Rohim, M. A. A. Mohamed, Housni El Saied, Hamdy A. Z. Hussein, A. Abdelkhalek

**Affiliations:** 1 Cellulose & Paper Department, National Research Centre, El-Buhouth St., Dokki, Cairo, 12622 Egypt; 2 Phytochemistry and Plant Systematics Department, National Research Centre, Dokki, Cairo, 12622 Egypt; 3 Horticultural Crops Technology Department, National Research Centre, Cairo, 12622 Egypt; 4 Fruit Handling Department, Horticulture Research Institute, Agricultural Research Center, Giza, 12619 Egypt; 5 Horticulture Department, Faculty of Agriculture, Fayoum University, Fayoum, 63514 Egypt

**Keywords:** Active packaging, Fruit quality, *Malus domestica* var. Anna, Nanochitosan, Packaging materials

## Abstract

Storage of economic fruits is a fatal economic and nutritional factor for most countries. Edible coating played a restricted role in this manner, forcing good usability with many limitations. In this work, Apple (*Malus domestica* var. Anna) was coated using bioactive nanopackaging films formulated to overcome the economic limitations and drawbacks of conventional coating. The formulated bioactive nanopackaging was based on nanochitosan prepared from shrimp shells and orange peel waste, which is used to produce nanocellulose (white part) and extract active ingredients (orange part). The formulated bioactive nanopackaging based on nanochitosan and nanocellulose (2:1) and orange peel waste extract with ratios of 1, 3, and 5% (w/w) based on nanochitosan dry weight and called **T3**, **T4**, and **T5**, respectively. Characteristics of bioactive nanopackaging films and their precursor materials were characterized physicochemically and topographically as well. The waste orange peel waste extract was characterized phytochemically. According to the orange peel extract, the formulated bioactive nanopackaging films observed antioxidant and antimicrobial activity. The results revealed that all treatments outperformed the control, especially treatments **T4** (1% Nano Chitosan + 1% Nano Cellulose + 3% Orange Peel Waste extract) and **T5** (1% Nano Chitosan + 1% Nano Cellulose + 5% Orange Peel Waste extract), in terms of fruit decay percentage (11.72 ± 11.4 C and 12.33 ± 10.83 C, respectively), weight loss (3.81 ± 2.29B and 3.77 ± 2.22B, respectively), TSS/acidity (17.07 ± 1.14 A and 16.77 ± 1.18 A, respectively), fruit firmness (12.83 ± 1.19B and 13.48 ± 0.91 A, respectively), total sugars (7.98 ± 0.21 A and 8.21 ± 0.29 A, respectively) and total anthocyanin (0.21 ± 0.03B and 0.25 ± 0.05 A, respectively).

**Supplementary Information:**

The online version contains supplementary material available at 10.1186/s40643-025-00890-9.

## Graphical Abstract

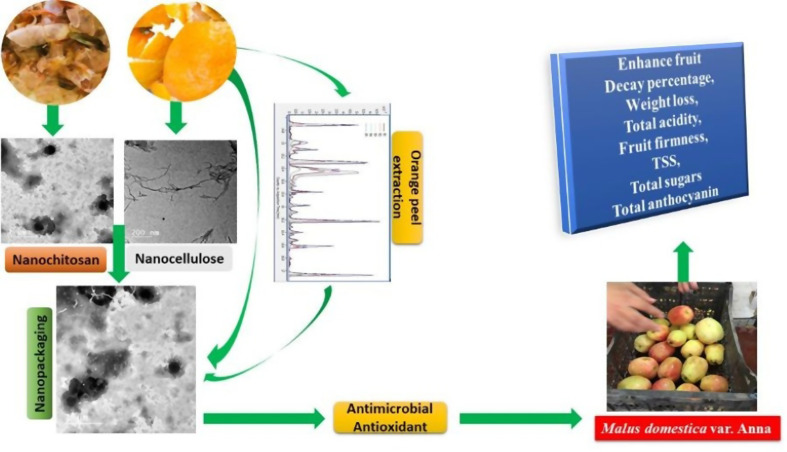

## Introduction

Package containers account for 23% of the materials landfilled worldwide, some of which are food-related (Podd 2022). Some food process wastes are economical with zero value and have a high natural active component content, including food process waste such as oranges (Maqbool et al. 2023). Food processing waste management has many drawbacks, including recycling, reuse, incorporation into strategic industries, and negative environmental impact (Peng et al. 2023). On the other hand, the packaging of fruit is usually based on fossil-derived plastic packaging materials (El-Naggar et al. 2022). These wastes typically hurt the environment and population. Unfortunately, packaging materials based on plastics have numerous drawbacks; namely, they are non-biodegradable, non-biocompatible, toxic, and impermeable. Alongside the quality of raw materials and processing equipment, food packaging is an indispensable aspect of the food industry. Food packaging sector is a crucial component of the packaging industry, necessitating adherence to stringent quality and safety standards and compliance with governmental rules and policies to achieve market success (Elsayed et al. 2022). Alternative strategies for packaging materials usually depend on the high price of neat materials offered and overcoming the drawbacks of plastic materials. Otherwise, active packaging materials still need more research to fit with the food packaging, especially for fresh fruits (Weber Macena et al. 2021).

Indeed, the bioactive components could be achieved from waste materials, especially the peels of fruits, which contain many active ingredients at low prices. In this regard, orange peels constitute the predominant waste regarding volume and usability within the orange sector. Approximately 20% of an orange is peel. The orange peelcontains 23% sugar, 22% cellulose, 25% pectins, and 11% hemicellulose (Ayala et al. 2021). Citrus peel is a significant source of essential oils, particularly limonoids, utilized in manufacturing fragrances and various cosmetics. Limonoids possess several biological properties, including antiviral, antifungal, and antibacterial actions (Ibrahim et al. 2021). Citrus peel can generate valuable products such as biogas, ethanol, or volatile flavoring chemicals, and extract, isolate, and purify bioactive molecules, essential in developing health-oriented goods. Citrus peel has numerous compounds with intriguing features that can serve as natural additions across various industries. These include pectin extracted by acid treatment and complete dietary fiber derived from mechanical processing (Buljeta et al. 2023). Citrus peel is a significant source of natural phenolic chemicals exclusive to citrus, particularly the distinctive flavanone glycosides, primarily naringin, hesperidin, rutin, and neohesperidin. The total phenol content of orange peel varies from 1.13 to 7.30 g/100 g. Citrus flavonoids possess health-related effects, including antioxidant, anticancer, antiviral, and anti-inflammatory actions (Saini et al. 2022).

Apple fruits *(Malus domestica var. Anna*) exhibit significant variation in bioactive compounds, including polyphenols, flavonoids, vitamins, and pigments, which contribute to their high nutritional value. Regrettably, these components significantly diminish during post-harvest periods (Sümbül et al. 2025).

This may pertain to the proliferation of certain microorganisms on fruit surfaces during the post-harvest period, which generate mycotoxins and decompose phytochemicals (Awuchi et al. 2021). Furthermore, apple cultivar, environmental and agronomic conditions, harvest and food processing practices, and storage variables may also influence phytochemical degradation. These concerns are often mitigated by applying commercial waxes and synthetic chemicals such as thiabendazole (WW-TBZ) to fruits. Nonetheless, these compounds may induce hazardous adverse effects associated with cancer (He et al. 2024). The modern application of fruit coating techniques involves using natural polymers, such as chitosan (poly B-(1,4) N-acetyl-D-glucosamine), combined with natural additives derived from food processing and fruit peel waste (Hasanin et al. 2023a). Chitosan is the second most prevalent polysaccharide in nature, following cellulose. Besides being environmentally safe, it possesses excellent film-forming capabilities and antibacterial efficacy and has been endorsed as a GRAS food additive (Casalini and Giacinti Baschetti 2023).

Dual role strategies were included to reduce waste and prepare a new formula of bioactive packaging material for fruit packaging. Therefore, in this research, new formulations of bioactive nanopackaging coats based on cellulose from orange peel (white part) are loaded with active components (orange part). These formulae were collected using chitosan nanoparticles. These formulae offered antimicrobial, antioxidant, and low-humidity conditions, etc. These formulations coat Anna apples to extend the storage and quality properties.

## Materials and methods

### Materials

Shrimp waste was collected from local fish shops in Egypt. “Anna” apple fruits were purchased from a private farm in Giza City, Egypt. Apple trees (*Malus domestica* var. Anna) were 10 years old. Orange peel waste (*Citrus sinensis*) was collected from a local juice market in Giza, Egypt. All chemical, reagent, and microbial media were purchased from LoBa Chem, India, in analytical grade and used without any previous purification.

### Methods

#### Deacetylation of shrimp waste

The following three processes isolate chitosan from shrimp waste: demineralization, deproteinization, and deacetylation. The shrimp waste was manually removed from the flesh after the obtained shrimp samples were cleansed entirely with water and dried in the oven at 70 ^o^C for 72 h. According to (Said Al Hoqani et al. 2020), shrimp samples were ground into powder (with a particle size of about 0.5–1 mm) and prepared for demineralization. Shrimp waste shell powder was added to a 45% sodium hydroxide solution in a 1:10 (*w/v*) ratio in a reaction vessel following (Mafosso-Tanto et al. 2024), and the reaction was under a nitrogen atmosphere. After the reaction, the product was filtered and rinsed with distilled water until a neutral pH was achieved. The resultant chitosan was dried in a vacuum oven for 24 h at the same temperature.

#### Nano Chitosan preparation

Nanochitosan was prepared according to our previous work (Shehabeldine et al. 2022). One gram of chitosan was dissolved in water with 1% acetic acid and 1% tripolyphosphate (TPP). Nanoparticles were spontaneously synthesized by introducing 1 mL of TPP aqueous solution into 10 mL of chitosan solution while maintaining steady stirring at room temperature for 30 min. The synthesized nanoparticles were isolated via centrifugation at 10,000 rpm for an hour, thereafter purified, dispersed in water, and lyophilized.

#### Extraction of nanocellulose

After scraping the orange layer, the internal layer of orange peels was collected. The white layer is a cellulose-rich layer that can be used after acid treatment (0.5 M HCl for 3 h at 100 ^o^C). The collected cellulose fibers were washed seven times with deionized water until neutralization, dried at 70 °C overnight, and then ground. Afterwards, the TEMPO oxidation was carried out according to our previous work (Shehata et al. 2024).

#### Extraction of the orange Peel active ingredients and its phenolic profiling

An ultrasonic water bath extracted the orange peel using ethanolic solvent (70%, *v/v*). The collected extract was concentrated using Rotavapor^®^ (Heizbad Hei-VAP, Heidolph, Germany), lyophilized (Christ, Osterode am Harz, Germany), and stored in the refrigerator (at -10 ^o^C) for further use. The phenolic profile of the orange peel waste extract was illustrated using high-performance liquid chromatography (HPLC) (Abdel-Haleem et al. 2024).

#### Formulation of the bioactive nanopackaging films

Nanochitosan/cellulose films were formulated in a 2:1 ratio with different concentrations of orange peel extract added in ratios of 1, 3, and 5% (w/w) based on nanochitosan, as shown in Table 1. These selected concentrations aim to investigate a potential dose-response association, commencing at a low concentration (1%) and progressively increasing to 5% to evaluate the effects of escalating extract levels (Chaiwarit et al. 2018; Terzioğlu et al. 2021). The formulated solution was stirred for one h at 1500 rpm at 70 ^o^C. The collected solution was sonicated using an ultrasonic probe for 10 min. and cast to form the bioactive packaging films.

**Table 1:** Abbreviation of bioactive films prepared in the study

Film content	Abbreviations
Control (dipping in water)	T1
1% Nano Chitosan	T2
1% Nano Chitosan + 1% Nano Cellulose + 1% Orange Peel Waste extract	T3
1% Nano Chitosan + 1% Nano Cellulose + 3% Orange Peel Waste extract	T4
1% Nano Chitosan + 1% Nano Cellulose + 5% Orange Peel Waste extract	T5

#### Characterization of bioactive nanopackaging films

Attenuated Total Reflectance-Fourier Transform Infrared Spectroscopy (ATR-FTIR) using the Spectrum Two IR Spectrometer from PerkinElmer, Inc., Shelton, USA. All spectra consist of 32 scans and possess a resolution of 4 cm − 1 across the wavenumber range of 4000 to 400 cm^− 1^. The high-resolution transmission electron microscope (HR-TEM), Model JEM2010 from Japan, was employed to assess the particle size and shape of samples. Dynamic light scattering (DLS) equipment (Santa Barbara, CA, USA) was employed to determine the average particle size distribution in nanometers and the average zeta potential in millivolts at 23 °C, using the 632.8 nm line of a HeNe laser at an angle of 13.9°.

#### Fruit quality and bioactive substances study

Fruits were cleaned, sorted, and graded. Then, the treatments were done by dipping fruits in the packaging film solution and removing all excess moisture. Fruits were stored at a temperature of 0 °C and a relative humidity of 85–90%. Physical and chemical characteristics of fruits were done fortnightly, including the decay percentage of total fruit count at the commencement of cold storage. The apple fruit weight loss (%) was individually labeled and weighed before starting cold storage (initial weight). Cold storage (one-month interval) was calculated at each sampling date according to Fathizadeh et al. (2021). Fruit firmness (Newton) was measured as (Ib/in^2^) on the two opposite sides at the tropic of the fruit after removing the peel, using an Effigi pressure tester (mod. Ft 327). The values of readings were converted to Newton unit (Ib/in^2^ × 4.448) since this unit is required for scientific writing (Nagy 2018).

Freshly prepared juice of apple fruits per each sample was extracted using an electric fruit juicer to assess the measurements, including total sugars (%), which were extracted and evaluated by using the phenol-sulphuric acid colorimetric method, according to (Akpomie et al. 2021). Total soluble solids (TSS) content as Brix was measured at 20 °C using a hand refractometer. Total acidity (%) was determined by titration with 0.1 N sodium hydroxide in the presence of a phenolphthalein indicator (Tomala et al. 2022). Vitamin C content was determined and expressed as mg L-ascorbic acid/100 ml fresh fruit juice using the method described by (Tomala et al. 2022). The anthocyanin content of apple fruits was determined according to (Ponder et al. 2022), and total chlorophyll content was determined according to Fuleki and Francis (Starowicz et al. 2020).

#### Polyphenolic and sugar profile analysis of fruits

The treated fruits were extracted *via* hydroalcoholic solvent (70% ethanol) using an ultrasonic water bath. The collected extracts were concentrated using Rotavapor^®^ (Heizbad Hei-VAP, Heidolph, Germany), lyophilized (Christ, Osterode am Harz, Germany), and stored in the refrigerator for further use.

#### Phytochemical study

The polyphenolic profile was determined using HPLC analysis, illustrating the phenolic structure’s kind and concentration (Abdel-Haleem et al. 2024). Also, the sugar structures were recognized using Gas chromatography-mass spectrometry analysis (GC-MS) after extract preparation (Emam et al. 2024).

#### Antimicrobial activity

Antimicrobial activity was studied against the most food-poisonous microorganisms. There are three Gram-negative bacteria *Escherichia coli*, Gram-positive bacteria *Staphylococcus aureus*), yeast *Candida albicans*, and *Aspergillus niger* using turbidimetric assay (Abdelhameed and Hasanin 2024; Ibrahim et al. 2023). The antimicrobial activity was calculated according to the blank sample of each microorganism as a percentage of inhibition, and the results were given as the mean ± standard deviation (SD).

#### Antioxidant activity

According to (Hassan et al. 2024), the antioxidant behavior of formulated films was measured by a radical scavenging ability using the DPPH method. The tested samples were prepared in different concentrations ranging from 1 to 12 g/dL to evaluate the antioxidant activity behavior of the bioactive nanopackaging films.

### Statistical analysis

The experiment was structured as a factorial design within a completely randomized block framework. Each treatment was duplicated thrice. An ANOVA analysis of the data was conducted utilizing the InfoStat statistical tool, version 2020, with the replicate as the random variable. Multiple comparisons of means were performed using the Duncan test with *p* = 0.5.

## Results and discussion

### Active packaging film characterizations

**Fig. 1:**
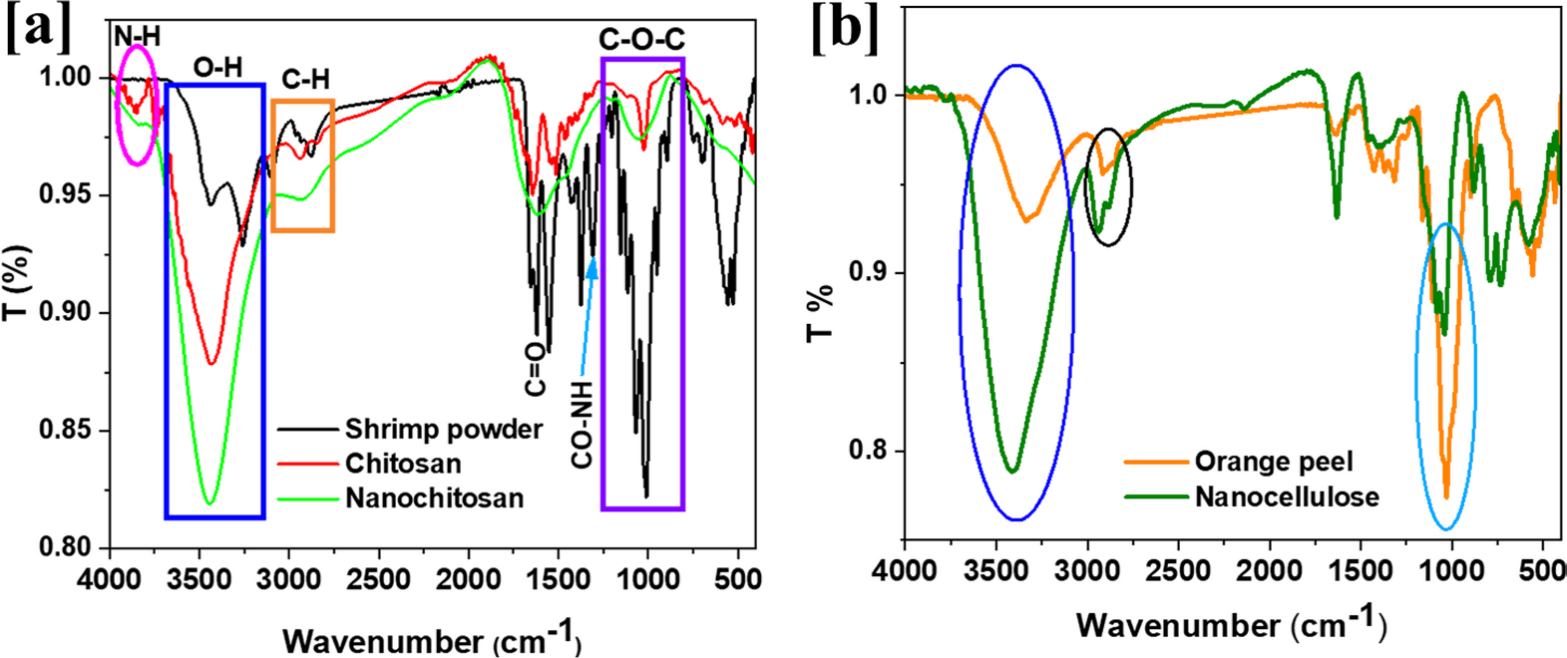
FTIR spectra of shrimp powder, chitosan, and nanochitosan (**a**) as well as orange peel conversion to nanocellulose (**b**)

Deacetylation of shrimp waste shells was carried out using sodium hydroxide. FTIR illustrated the shrimp conversion to nano chitosan and the orange peel white layer conversion to nanocellulose spectra in Fig. 1. Figure 1a demonstrates that the shrimp powder showed no clear bands in the blue area of more than 3431 cm˗1, which referred to the overlapping of NH and OH groups stretching vibrations. Moreover, shrimp powder’s C-H stretching vibration band was recorded at 3096 and 2865 (sharp) cm^˗1^. On the other side, extracted chitosan recorded a sharp band of NH at 3850 cm^˗1^ as well as OH at 3431 cm^˗1^ with two small bands reflecting CH at 2936 and 2826 cm^˗1^ (Madhu et al. 2022). In addition, the nanochitosan observed a low-intensity NH band at 3845 cm^˗1^ and a high-intensity OH band at 3432 cm^˗1^ (Kurniawidi et al. 2022). However, the carbohydrate band was affected by shifting to the red frequency area after powder processing as 1015, 1021, and 1040 cm^˗1^ for shrimp powder, chitosan, and nanochitosan, respectively. Furthermore, the diminution of the band at 1615 and 1304 cm^− 1^, respectively, attributed to the CO-NH bending vibration and the stretching of the C = O in the amide bond, shows that the deacetylation was successful (Aldila et al. 2020). The FTIR data were affirmed by the successful deacetylation of chitin to chitosan and the formulation of nanochitosan. On the other side, the white layer of orange peel (blank) and nanocellulose were presented in Fig. 1b.

### Topographical study

**Fig. 2:**
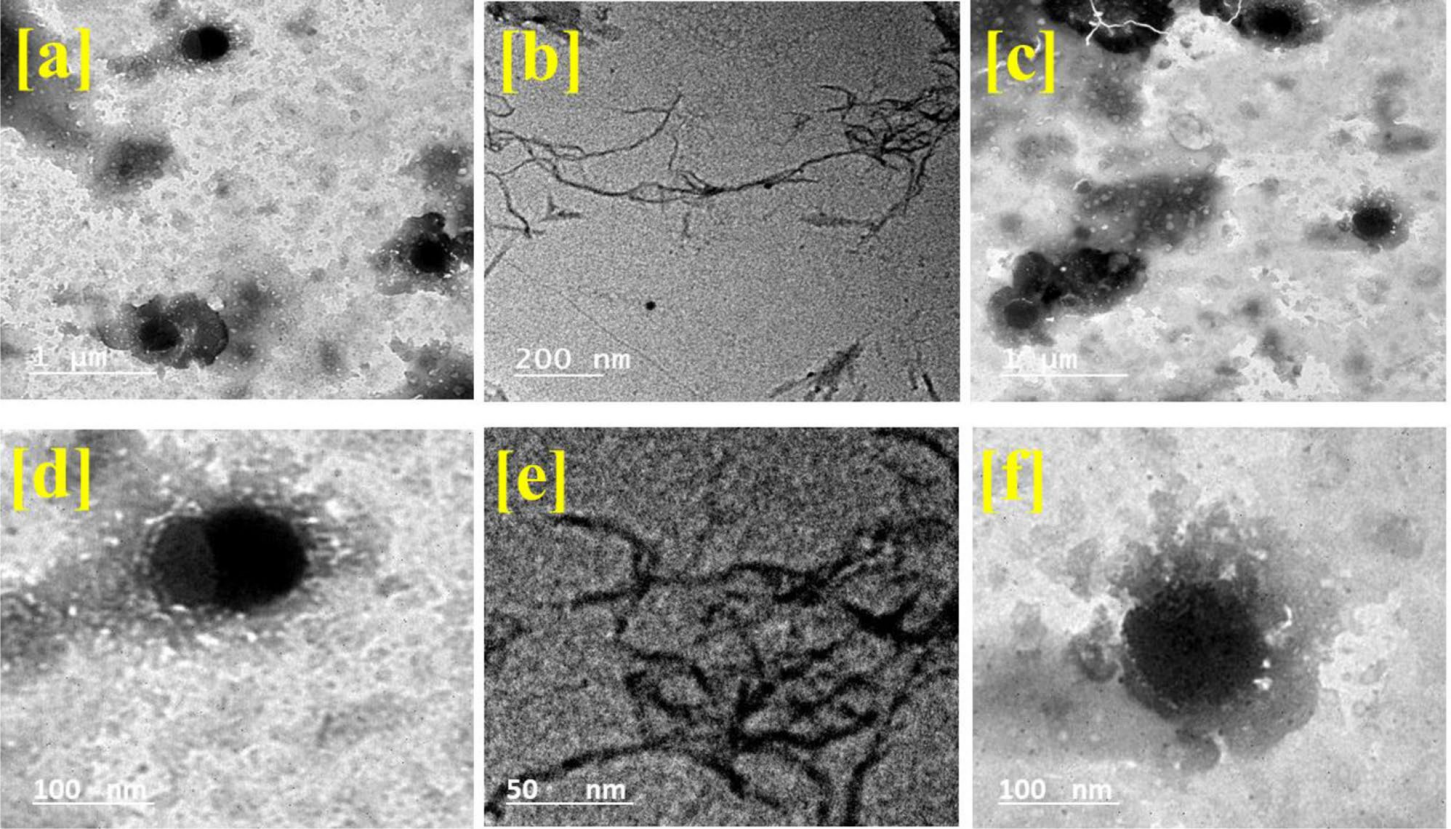
HR-TEM images of nanochitosan with low (**a**) and high (**d**) magnification, nanocellulose with low (**b**) and high (**e**) magnification, and bioactive nanopackaging film (3% orange peel extract) with low (**c**) and high (**f**) magnification

The HR-TEM images of nanochitosan, nanocellulose, and bioactive nanopackaging film (3% orange peel extract) are presented in Fig. 2. Nanochitosan was observed in Fig. 2a with low magnification as spheres with homogeneous shapes. A high magnification image of nanochitosan (Fig. 2d) shows the particle size of about 160 nm. This agrees with previous publications (Jesus et al. 2020; Quester et al. 2022). Furthermore, nanocellulose in a low magnification image (Fig. 2b) was presented as a network of nanofibers with a little tangle. The high magnification image (Fig. 2d) confirmed that the fibers have a length of about 1 mm and a width of about 3 nm (Hasanin et al. 2023b; Hasanin and Youssef 2022). However, the bioactive nanopackaging film image (low magnification) in Fig. 2c was observed as an integration between the morphological appearance of nanochitosan and nanocellulose. The high magnification image in Fig. 2f presents the tangle of nanocellulose and nanochitosan. In this context, the nanostructure of chitosan and cellulose, which was presented with HR-TEM images, as well as the nanostructure of bioactive nanopackaging film were also presented. Moreover, these observations affirmed the formulation of nanostructures and confirmed the DLS measurements.

**Fig. 3:**
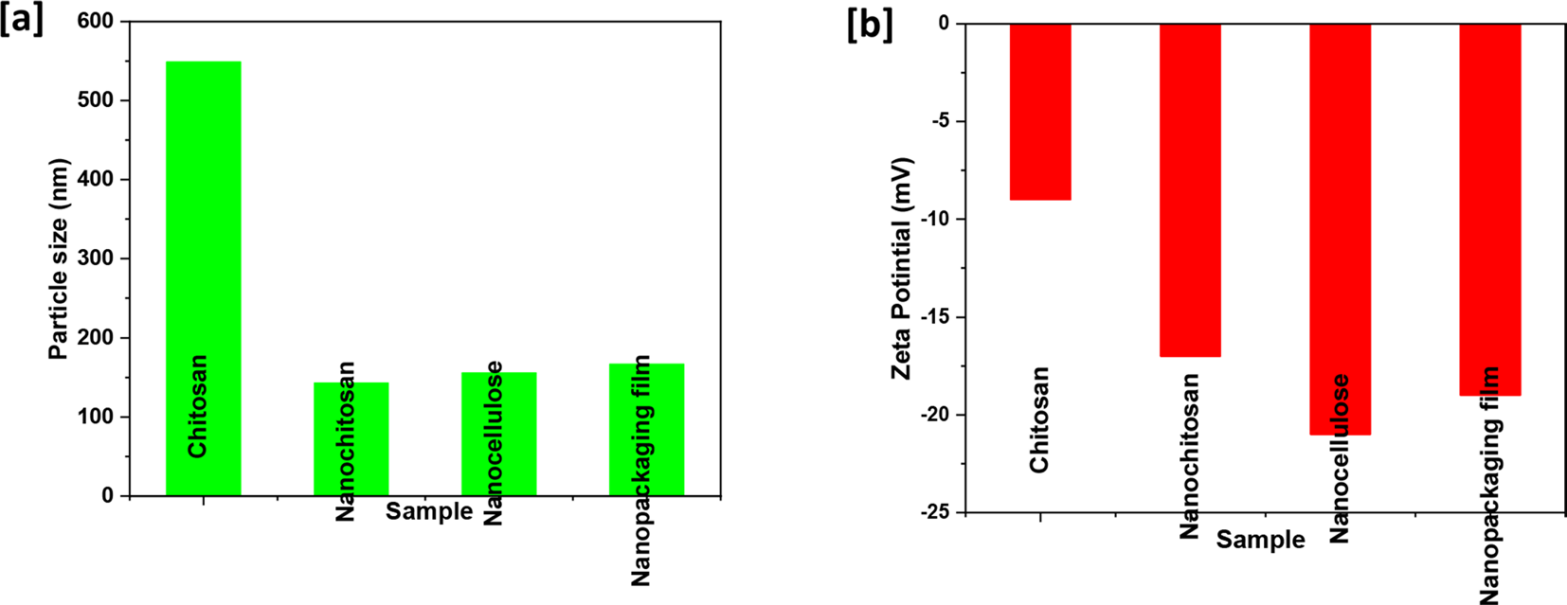
DLS measurements of chitosan, nanochitosan, nanocellulose, and bioactive nanopackaging film (3% orange peel extract). **a**) particle size distribution and **b**) average zeta potential

DLS measurements of nanochitosan, nanocellulose, and bioactive nanopackaging film (3% orange peel extract), including average particle size distribution, polydispersity index (PDI), and average zeta potential, were carried out. Figure 3 illustrates the average particle size distribution recorded as 489, 143, 156, and 167 nm for chitosan, nanochitosan, nanocellulose, and bioactive nanopackaging film, respectively. Moreover, the PDI was recorded as 0.36, 014, 0.19, and 0.23 index for chitosan, nanochitosan, nanocellulose, and bioactive nanopackaging film, respectively. In this context, particle size distribution and PDI values referred to low homogeneity of chitosan particles and excellent homogeneity for nanochitosan and nanocellulose that slightly decreased in bioactive nanopackaging film due to the particle size increase (Bi et al. 2021). On the other hand, the average zeta potential values of chitosan, nanochitosan, nanocellulose, and bioactive nanopackaging film were recorded as -9, -17, -21, and − 17 mV, respectively, affirming the particle size distribution results. These values referred to pure chitosan’s low stability and nanocellulose’s high stability. Meanwhile, nanochitosan shows acceptable stability, and bioactive nanopackaging film shows good stability (Dukhin and Xu 2020).

### Polyphenolic contents of orange peels

Citrus processing enterprises generate approximately 40 million tonnes of peel waste each year. Peel waste extract has many citrus polyphenols that may be utilized in pharmaceuticals (Khan et al. 2021). Flavanones, recognized as vital nutrients for preserving health and adding to the fruit’s aroma, make up most of these polyphenols (Silva et al. 2020). Citrus plants have different levels of bioactive polyphenols according to their species, cultivar, edaphoclimatic conditions, and maturation stage. It is generally understood that citrus fruits do not contain polyphenols in exact amounts (Shah et al. 2024; Musumeci et al. 2020). The HPLC profile of orange peel waste extract exposed to various polyphenol concentrations is presented in Table 2. Catechin, Quercetin, and Ferulic acid showed the highest concentration in the extract, while Kaempferol showed the lowest.

**Table 2:** Illustrated polyphenol contents (µg/g) of orange (*Citrus sinensis*) fresh peels

No.	t_*R*_ (min)	Metabolites	Conc. (µg/g)
1	3.59	Gallic acid	808.68
2	4.26	Chlorogenic acid	794.26
3	4.51	Catechin	3108.41
4	5.52	Methyl gallate	945.86
5	5.96	Caffeic acid	160.45
6	6.46	Syringic acid	316.43
7	6.98	Rutin	403.47
8	7.3	Ellagic acid	409.33
9	8.76	Coumaric acid	135.40
11	9.825	Ferulic acid	1986.36
12	10.52	Naringenin	218.46
13	11.959	Rosmarinic acid	764.02
15	17.443	Quercetin	2424.26
17	20.711	Kaempferol	21.12

***t***_**R**_: retention time, **Conc**: concentration

### Fruit quality and bioactive substances study

#### Fruit juice acidity

**Fig. 4:**
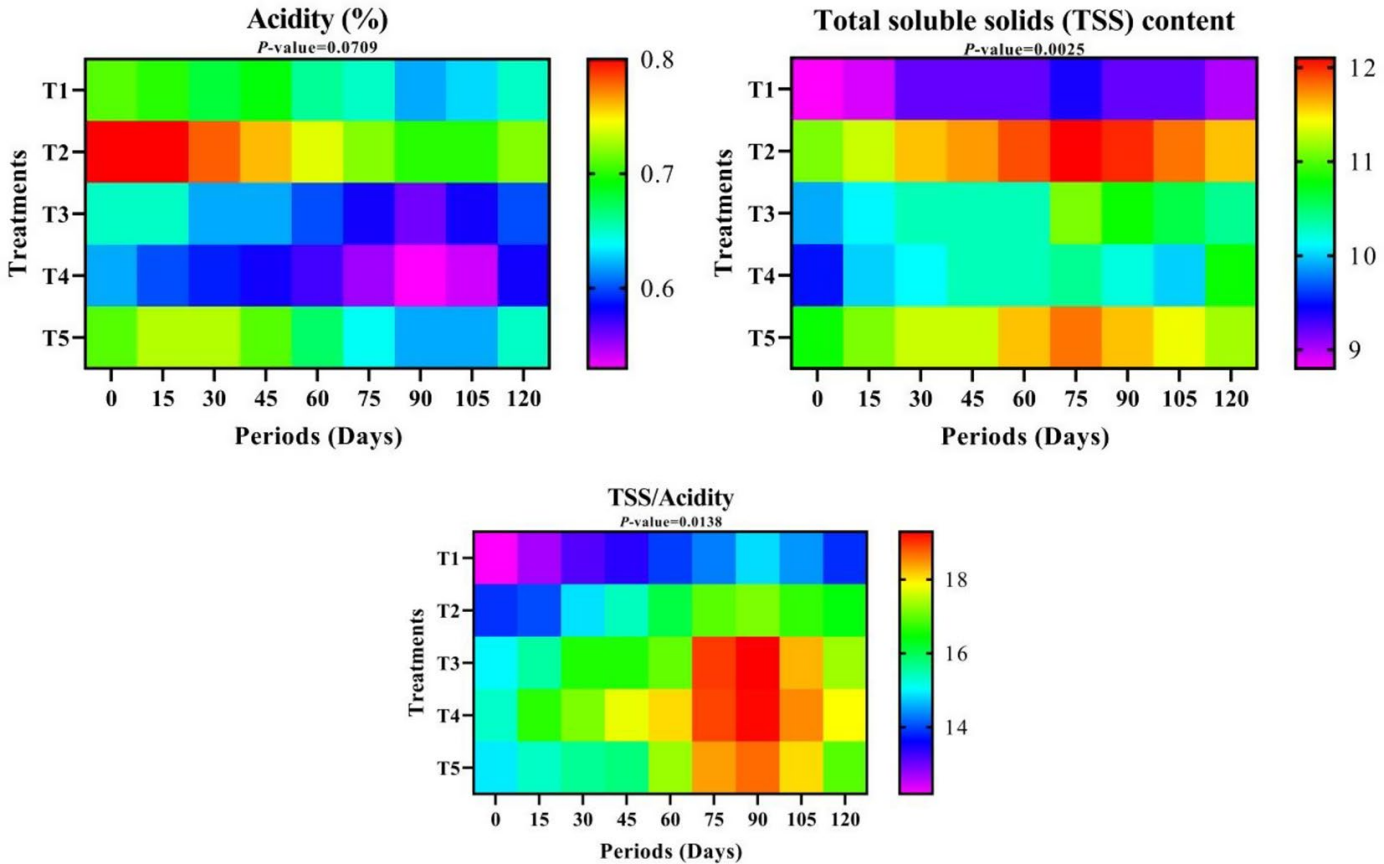
Total acidity (%), Total soluble solids (TSS), and TSS/Acidity over different storage periods of “Anna” apple cv. influenced by some packaging materials. T1 = Control (dipping in water), T2 = 1% Nano Chitosan, T3 = 1% Nano Chitosan + 1% Nano Cellulose + 1% Orange Peel Waste extract, T4 = 1% Nano Chitosan + 1% Nano Cellulose + 3% Orange Peel Waste extract, T5 = 1% Nano Chitosan + 1% Nano Cellulose + 5% Orange Peel Waste extract. The mean values are significant with *p ≤* 0.05

Regarding the differences between treatments, the lowest fruit juice acidity percentage resulted from T4, followed by T3, while the highest fruit juice acidity value appeared in T2. As for the storage period, the fruit juice acidity decreased over time until it reached its lowest level after 90 days of cold storage. However, it began to increase again when cold storage lasted for 105 and 120 days, as shown in Table S-1 and represented in Fig. 4.

#### Total soluble solids (TSS) content

Figure 4 and Table S-1 illustrate the total Soluble Solids (TSS) content, as T2 achieved the best TSS value, followed by T5, while the lowest values were observed when the fruits were coated with T3. The TSS took a different path in the cold storage period, as the TSS values were low at zero time, then increased with increasing storage time until they reached the highest value at 75 days of cold storage, and then gradually decreased again until they reached the lowest value at 120 days of cold storage. Regarding the relationship between treatments and storage periods, it was observed that the highest values of TSS were when fruits were coated with T2 for 75 and 90 days, respectively, while T3, at zero time and 15 days of cold storage, showed the lowest values, respectively.

#### TSS/Acidity

The results in Table S-1 and Fig. 4 clarified that all treatments outperformed the control concerning TSS/Acidity, especially T3 and T4. However, according to the storage periods, it was noted that TSS/Acidity increased over time until it reached the peak at 90 days, then decreased until it reached 16.54 after 120 days of cold storage. The interaction between treatments and periods showed that T3 outperformed when fruits were stored for 90 and 75 days, respectively. The lowest TSS/Acidity was in T1 at zero time and 15 days of cold storage, respectively.

#### Fruit decay

**Fig. 5:**
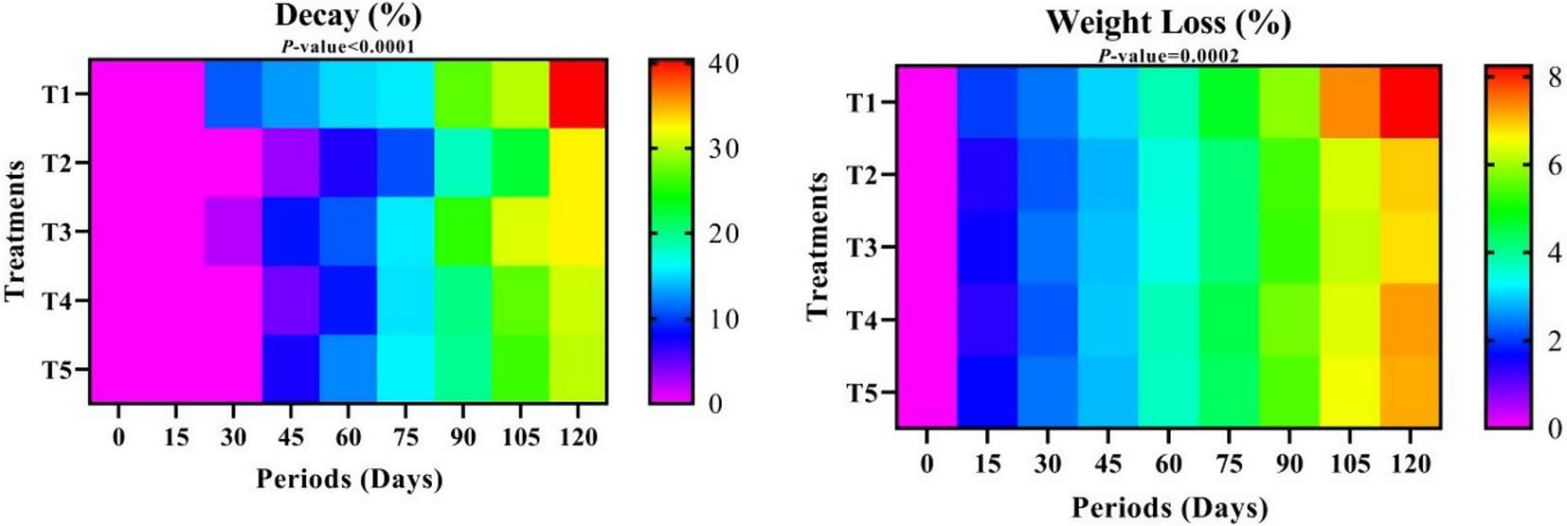
Decay (%) and Weight Loss (%) over different storage periods of “Anna” apple cv. influenced by some packaging materials. T1 = Control (dipping in water), T2 = 1% Nano Chitosan, T3 = 1% Nano Chitosan + 1% Nano Cellulose + 1% Orange Peel Waste extract, T4 = 1% Nano Chitosan + 1% Nano Cellulose + 3% Orange Peel Waste extract, T5 = 1% Nano Chitosan + 1% Nano Cellulose + 5% Orange Peel Waste extract. The mean values are significant with *p ≤* 0.05

Regarding post-harvest coating treatments’ impact on fruit decay rates, T2 showed a distinct superiority, followed by T4. The highest levels of fruit degradation were observed in the T1 (water dipping) and T3 treatments, respectively (Fig. 5 and Table S-2). The post-harvest cold storage duration influenced all treatments; the fruits remained unaffected for the initial fifteen days, but after one month, several fruits exhibited signs of deterioration, especially in T1 and T3 (11.31 ± 2.93j and 2.30 ± 1.0mn, respectively). A direct correlation existed between the duration of storage and the percentage of fruit degradation, peaking after 120 days of cold storage. According to the interaction between post-harvest treatments and storage durations, the minimal rate of fruit decay after the initial fifteen days of cold storage was observed in treatments T2, T4, T5, and T3 for 30 days, respectively. In contrast, the highest decay rates were noted in treatment T1 (water dipping) after 120 days.

#### Fruit Weight loss

According to the data in Table S-2, all coating treatments surpassed the control treatment (T1) in terms of percentage of fruit weight loss, with T4 exhibiting the lowest rate, followed by T3, while T1 displayed the highest percentages. The weight loss of fruit progressively escalated during post-harvest cold storage, peaking at 120 days of storage. The interaction between treatments and storage durations indicated that the lowest values at baseline across all treatments occurred with the T4 treatment after 15 days of cold storage, followed by T2 during the same interval. The most significant proportion of fruit weight loss, accompanied by a notable increase, was observed in T1, T4, T5, and T3 treatments after 120 days of cold storage.

##### Total chlorophyll content

The treatment T1 came out on top regarding the fruit total chlorophyll content, followed by T1, while T5 was the lowest compared to the rest of the treatments. Regarding cold storage periods, the fruit total chlorophyll content decreased over time, reaching a minimum of 120 days of cold storage. The interactions between coating treatments and storage periods took a different path, as T2 and T1 (dipping in water) treatments gave the highest values for fruit chlorophyll content after 15 days of cold storage, respectively, if we ignore the first period of cold storage (zero time), which gave the lowest values in all treatments. The treatment T5 for 120, 105, 90, 75, and 60 days of cold storage achieved the lowest values for fruit total chlorophyll content, respectively (Table S-3).

##### Total anthocyanin content

**Fig. 6:**
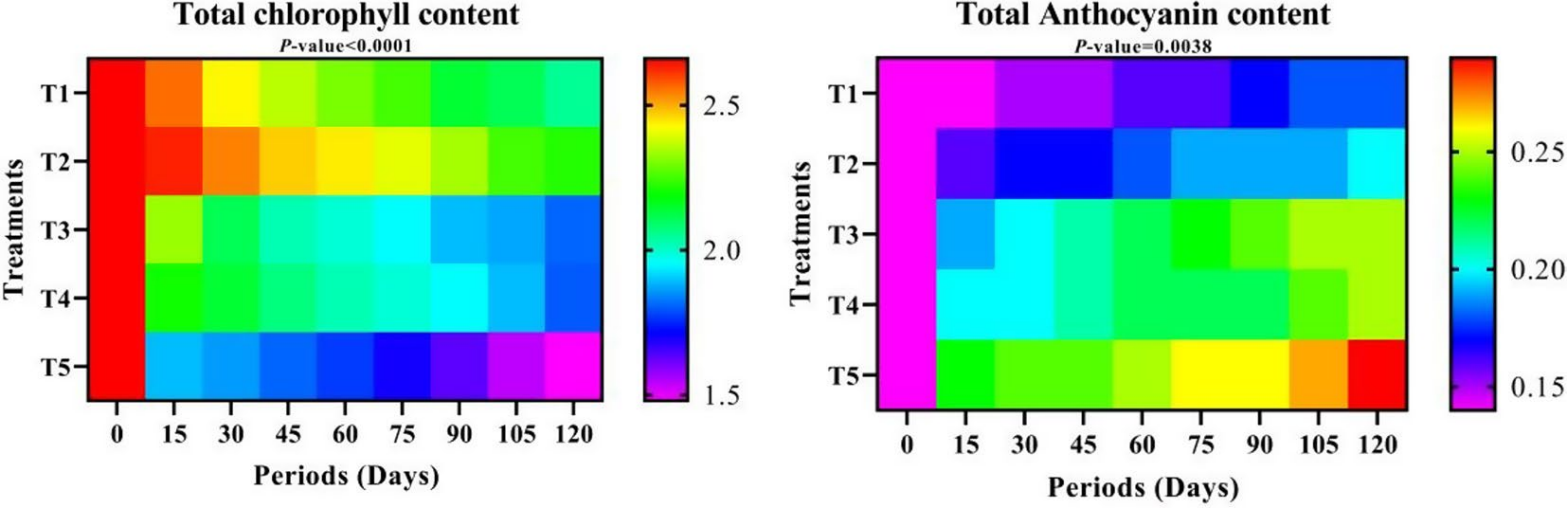
Total chlorophyll (mg g-1 FW) and Anthocyanin content over different storage periods of “Anna” apple cv. influenced by some packaging materials. T1 = Control (dipping in water), T2 = 1% Nano Chitosan, T3 = 1% Nano Chitosan + 1% Nano Cellulose + 1% Orange Peel Waste extract, T4 = 1% Nano Chitosan + 1% Nano Cellulose + 3% Orange Peel Waste extract, T5 = 1% Nano Chitosan + 1% Nano Cellulose + 5% Orange Peel Waste extract. The mean values are significant with *p ≤* 0.05

Let’s look at the effect of treatments on anthocyanin content in Table S-3 and Fig. 6. The highest anthocyanin content was in the T5 treatment, followed by T4 and T3 treatments, respectively, while the lowest was in the T1 treatment. Unlike total chlorophyll content, total anthocyanin content increased over time during cold storage, starting from zero time up to 120 days. The highest values of anthocyanin content were when the fruits were coated with T5 for 120 and 105 days, respectively. If we ignore the first period (zero time) in all treatments, which represented the lowest values, the lowest values after that were in the T1 treatment for 15 days of cold storage.

#### Total sugar content

**Fig. 7:**
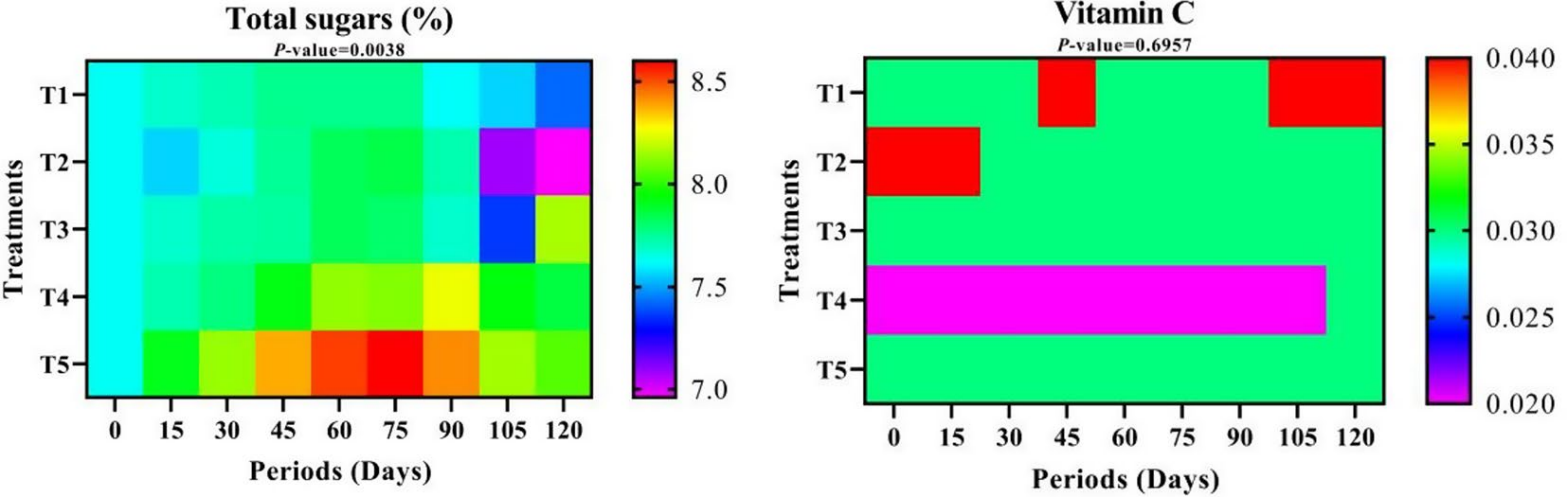
Total sugars (%) and Vitamin C content (mg /100 ml juice) over different storage periods of “Anna” apple cv. influenced by some packaging materials. T1 = Control (dipping in water), T2 = 1% Nano Chitosan, T3 = 1% Nano Chitosan + 1% Nano Cellulose + 1% Orange Peel Waste extract, T4 = 1% Nano Chitosan + 1% Nano Cellulose + 3% Orange Peel Waste extract, T5 = 1% Nano Chitosan + 1% Nano Cellulose + 5% Orange Peel Waste extract. The mean values are significant with *p ≤* 0.05

Although there were no significant differences between the treatments in Fig. 7 and Table S-4, T5 achieved the highest value of total sugar content in fruits, followed by T4. In contrast, the lowest values appeared in treatment T2 and T1 (dipping in water), respectively. Total sugar content in fruits followed a similar path to TSS content concerning periods of cold storage, as the lowest value was at zero time and then gradually increased until it reached the highest value at 75 days, then decreased again until it reached the second lowest value at 120 days of cold storage. According to the interaction between treatments and storage periods, it was observed that the highest values of total sugar content were when fruits were coated with T5 for 75 and 60 days, respectively, while T2 treatment at 120 and 105 days showed the lowest values, respectively.

#### Total vitamin C content

Data in Table S-4 and Fig. 7 revealed that T1, T2, and T3 treatments showed superiority in fruit vitamin C content, respectively, while the lowest values appeared when the fruits were coated with T4 material. Vitamin C content in fruits decreased over time during cold storage, starting from zero and up to 90 days, and then increased again until it reached 0.031 at 120 days, although the results were not significant.

#### Fruit firmness

**Fig. 8:**
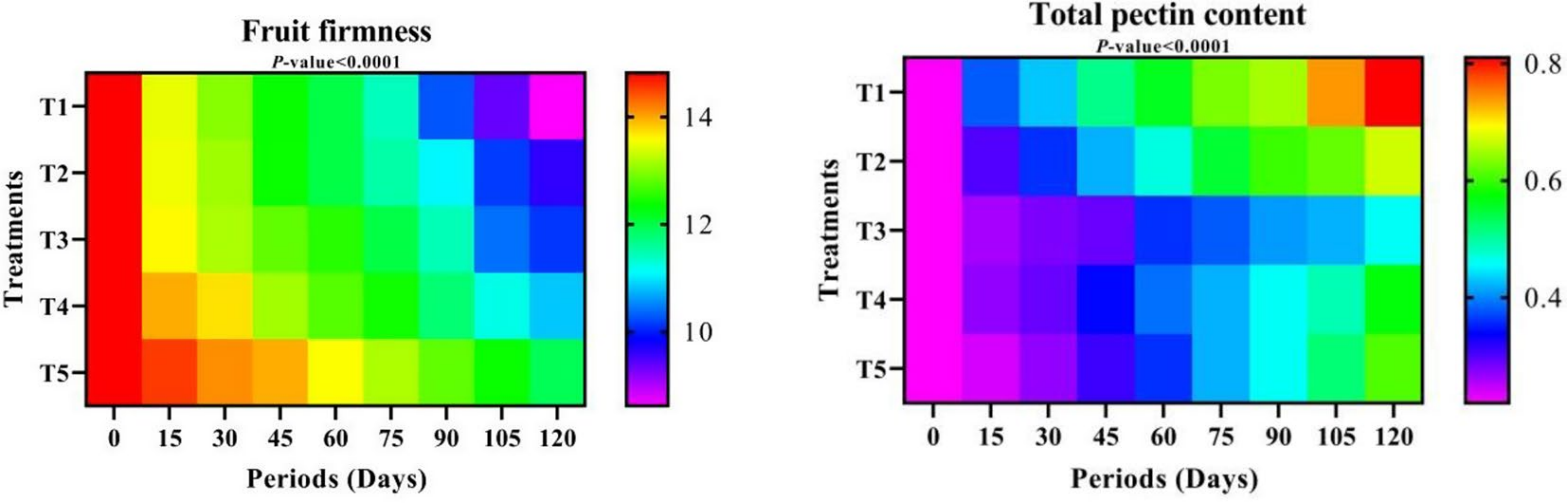
Fruit firmness and Total pectin content over different storage periods of “Anna” apple cv. influenced by some packaging materials influence apple fruits. T1 = Control (dipping in water), T2 = 1% Nano Chitosan, T3 = 1% Nano Chitosan + 1% Nano Cellulose + 1% Orange Peel Waste extract, T4 = 1% Nano Chitosan + 1% Nano Cellulose + 3% Orange Peel Waste extract, T5 = 1% Nano Chitosan + 1% Nano Cellulose + 5% Orange Peel Waste extract. The mean values are significant with *p ≤* 0.05

T5 showed a noticeable increase in fruit firmness compared to the rest of the treatments, while T1 came in last. Like fruit decay, fruit firmness decreased to a minimum at 120 days of cold storage, which applies to storage periods. According to the interaction of treatments and periods, T2, T5, T3, and T4 treatments at zero time recorded the highest rate of fruit firmness. At the same time, T1 gave the lowest fruit firmness after 120 and 105 days of cold storage, respectively (Fig. 8 and Table S-5).

#### Total pectin content

The T1 treatment (dipping in water) achieved the highest values regarding fruits’ total pectin content, followed by the T2 treatment, while the lowest values appeared in T4, which was equal to the T5 treatment. During cold storage, the fruits’ total pectin content increased from zero to 120 days. According to the interaction of treatments and storage periods, it was observed that the highest values of total pectin content were in T1 treatment after 120 and 105 days of cold storage, respectively, while the lowest values were at zero time in all treatments, followed by T5 treatment after 15 days of cold storage (Table S-5).

It is noted from the above that nano-packaging films has improved fruit quality and bioactive substances' content as shown below from brevious studies. Bioactive nanopackaging film coats effectively reduced fruit weight loss and titratable acidity, and enhanced total soluble solids and bioactive compounds in fruits and vegetables during storage (Oladzadabbasabadi et al. 2023). Bioactive nanopackaging film coats have improved fruit quality, permeability, and storability, including strawberries (Nguyen and Nguyen 2020a) and papaya (Pandey et al. 2022). The bioactive nanopackaging film coats provide higher barrier properties to the interior gas atmosphere and have been optimized for fruit permeability (Hashem et al. 2023). The storage life of coated fruits was much longer than that of the control. This might be due to chitosan’s barrier properties, protecting the fruit against physical, chemical, and biological deterioration (Wang et al. 2024). (Rodriguez et al. 2021) found that coated apples had a better appearance and had less dehydration during storage via reduced moisture loss from the fruit, retaining their texture. The bioactive nanopackaging film coat layers act as physical barriers to prevent drying and shriveling, reducing fruit weight loss (Nguyen and Nguyen 2020b). Bioactive nanopackaging film coating has been shown to reduce the respiration rate of fresh fruit and vegetables, resulting in delayed ripening (Shan et al. 2023). Total soluble solids increased in all samples under cold storage until 75 days, after which TSS decreased. Sucrose hydrolysis might explain this decline during fruit respiration (Brizzolara et al. 2020). The polyphenolic concentrations of uncoated samples rose with time, while those of coated samples varied over storage. This might be related to the breakdown of the cell structure during fruit senescence (Nguyen and Nguyen 2020a) or the formation of phenolic compounds during non-enzymatic processes (De Bruno et al. 2023). Total anthocyanin fluctuations during storage may also be connected with variations in total polyphenolic content (Nguyen and Nguyen 2020b). Moreover, nanocellulose has been found to significantly increase the shelf life of fruit, including persimmons and grapes (Chen et al. 2022). Furthermore, incorporating nanocellulose into packaging materials has enhanced mechanical properties, water barrier performance, and thermal stability (Zhang et al. 2022). The vapor pressure differential between the fruit tissue and the surrounding environment determines water loss from fruit. Edible coatings can build a layer on the fruit peel that reduces water loss, protects it from mechanical damage and microbial assault, and seals minor wounds (Pham et al. 2023). Edible coatings based on carboxymethyl cellulose and gum Arabic improved Acid Lime fruit quality and shelf life (Beheiry et al. 2023). 

### Phytochemical study

#### Polyphenolic contents of fruit

In addition, five different extracts of apple samples were investigated for their polyphenolic contents, as shown in Table 3. It was clear that the T4 treatment showed the highest gallic acid, catechin, vanillin, ferulic acid, naringenin, and rosmarinic acid contents. While the chlorogenic acid, coumaric acid, and quercetin contents were the most predominant in T1. In addition, T3 was characterized by methyl gallate and ellagic acid content.

**Table 3:** A polyphenolic content (µg/g) of five different apple (*M. domestica* var. Anna) extracts.

No.	t_*R*_ (min)	Metabolites	T1	T2	T3	T4	T5
1	3.59	Gallic acid	0.22	0.15	0.23	0.50	0.23
2	4.26	Chlorogenic acid	0.97	0.55	0.58	0.58	0.43
3	4.51	Catechin	0.09	0.02	0.02	0.20	0.03
4	5.52	Methyl gallate	0.13	0.11	0.14	0.07	0.12
5	5.96	Caffeic acid	0.05	0.07	0.04	0.00	0.04
6	6.98	Rutin	0.01	0.00	0.02	0.01	0.03
7	7.3	Ellagic acid	0.02	0.02	0.19	0.02	0.11
8	8.76	Coumaric acid	0.02	0.01	0.01	0.01	0.01
9	9.16	Vanillin	0.03	0.01	0.02	0.06	0.02
10	9.82	Ferulic acid	0.20	0.06	0.14	0.28	0.10
11	10.52	Naringenin	0.21	0.11	0.16	0.30	0.11
12	11.95	Rosmarinic acid	0.09	0.10	0.07	0.15	0.08
13	17.44	Quercetin	0.02	0.00	0.00	0.00	0.00

**T**: Treatment, ***t***_**R**_: retention time

In apple fruits and extracts, phenolic chemicals have a crucial role in their sensory qualities, nutritional value, metabolic impact, and stability over time. The distribution and abundance of certain polyphenols in different apple treatments are influenced by post-harvest processing, maturity stages, packing, and variety traits (Table 3). Phenolic chemicals have a major impact on apples’ color, astringency, bitterness, and taste. Apple peel and immature fruit are frequently associated with astringency and bitterness, which may be attributed to catechin, quercetin, and chlorogenic acid (Boyer and Liu 2004), especially when combined with sugars and esters, vanillin adds pleasing fragrance characteristics. Ferulic and coumaric acids are linked to enzymatic browning events that impact the appearance of processed apples (Arnold and Gramza-Michałowska 2022). Rosmarinic acid and naringenin, which are less prevalent in apples, may add delicate herbaceous or citrusy flavors to high-phenolic samples like T4, boosting their flavor complexity.

T1 and T4, which have higher levels of quercetin, chlorogenic acid (T1), and catechin, ferulic acid, and vanillin (T4), are anticipated to have stronger flavors and possibly more bitterness (Gaudette and Pickering 2012; Kumar et al. 2024). This makes them helpful for choosing particular organoleptic profiles or in natural flavor compositions.

As apples mature and stored, their phenolic content varies dynamically. While quercetin and catechins may build up in the peel as a protective mechanism against oxidative stress, chlorogenic acid tends to decrease throughout ripening (Awad and de Jager 2000). T3 may be derived from apples that have been stored under oxidative or prolonged circumstances because methyl gallate and ellagic acid, which are contained in T3, frequently increase in response to stress or oxidation. Because gallic acid and ferulic acid degrade enzymatically during storage, their presence in T4 may suggest early harvest or minimum processing (Aécio 2014; McMurrough et al. 1996; Gao et al. 2019). As a result, T1 might be a sample that has been more matured and kept, whereas T4 might be a fresher or early-stage extract that is useful for high-antioxidant goods.

Generally, treatments high in coumaric acid, quercetin, and chlorogenic acid may account for the mature or peel-rich sample’s strong antioxidant defense. Hydrolysable tannins are abundant in treatments that contain methyl gallate and ellagic acid, which may have oxidized or over-ripened the sample. While, the treatments rich in multifunctional phenolics like gallic acid, offers the greatest therapeutic potential, especially in metabolic and health applications, antioxidant, antimicrobial, and potential anti-cancer agent. Also, the treatments high in rosmarinic acid, gallic acid, catechin, vanillin, ferulic acid, naringenin, and broad-spectrum phenolics suggest fruit that is fresh or has been maintained to retain its antioxidant content.

#### Sugar profile of fruit

**Fig. 9:**
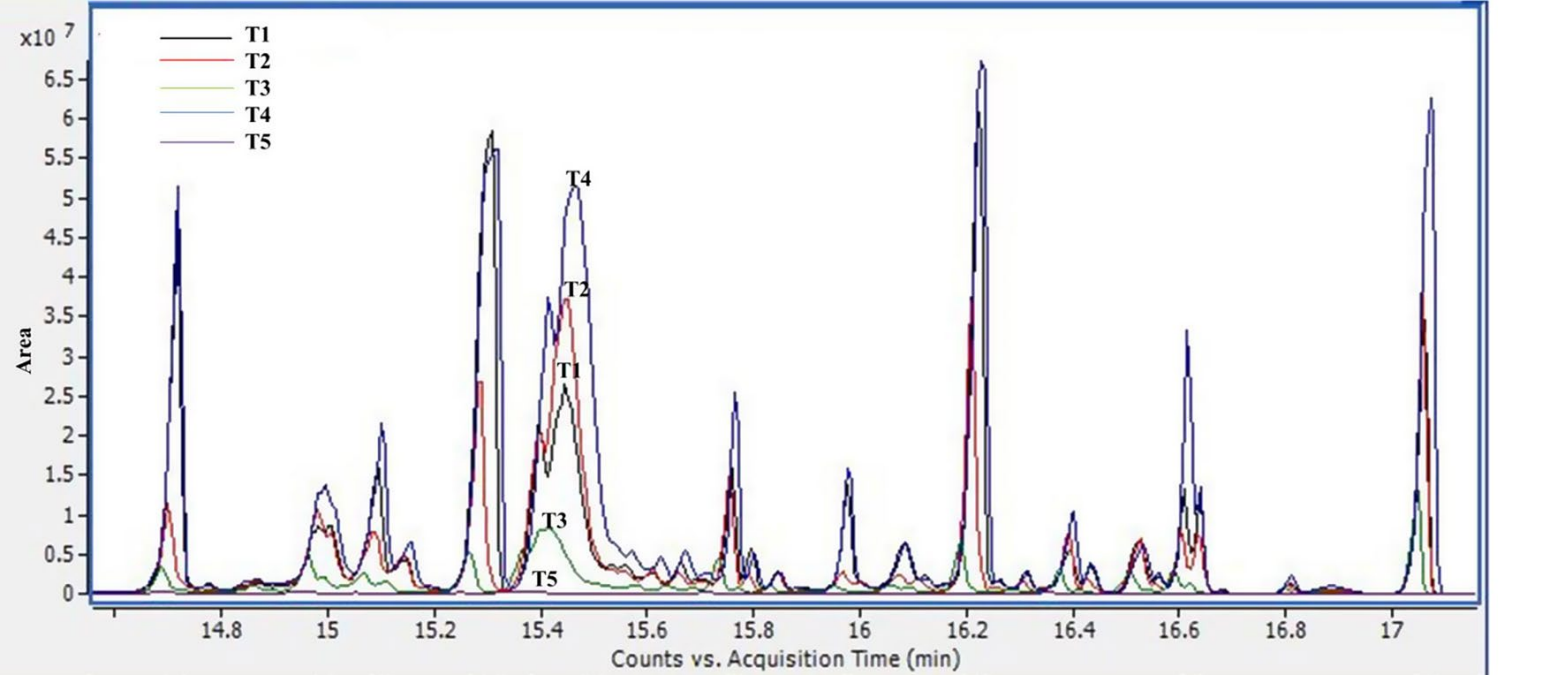
Total ion GC-MS Chromatograms of apple fruit (*M. domestica* var. Anna) extracts [T(1–5)]

The silylation method was used to illustrate the existing sugars and their percent area to differentiate between the different apple samples and the changes in chemical components of the stored apple fruits. All samples illustrated the same pattern of the identified sugars (Table 4) with different percent areas, such as T4, which showed the highest areas of sugars, as mentioned in Fig. 9.

**Table 4:** The identified silylated sugars of *M. domestica* Var. Anna. T(1–5) extracts using **GC-MS**

No.	t_*R*_ (min)	Name	Formula
1	8.24	Dihydroxyacetone, 2TMS derivative	C_9_H_22_O_3_Si_2_
2	14.30	D-Lyxose, 4TMS derivative	C_17_H_42_O_5_Si_4_
3	15.09	D-(-)-Fructofuranose, pentakis(trimethylsilyl) ether (isomer 1)	C_21_H_52_O_6_Si_5_
4	15.30	D-(-)-Fructofuranose, pentakis(trimethylsilyl) ether (isomer 2)	C_21_H_52_O_6_Si_5_
5	15.39	D-Psicofuranose, pentakis(trimethylsilyl) ether (isomer 2)	C_21_H_52_O_6_Si_5_
6	15.53	D-(-)-Ribofuranose, tetrakis(trimethylsilyl) ether (isomer 1)	C_17_H_42_O_5_Si_4_
7	15.55	D-(-)-Ribofuranose, tetrakis(trimethylsilyl) ether (isomer 1)	C_17_H_42_O_5_Si_4_
8	15.60	D-(-)-Ribofuranose, tetrakis(trimethylsilyl) ether (isomer 1)	C_17_H_42_O_5_Si_4_
9	15.66	Arabinofuranose, 1,2,3,5-tetrakis-O-(trimethylsilyl)-	C_17_H_42_O_5_Si_4_
10	15.75	Methyl.α.-D-glucofuranoside, 4TMS derivative	C_19_H_46_O_6_Si_4_
11	15.79	Methyl.α.-D-glucofuranoside, 4TMS derivative	C_19_H_46_O_6_Si_4_
12	15.97	D-Fructose, 1,3,4,5,6-pentakis-O-(trimethylsilyl)-, *O*-methyl oxime	C_22_H_55_NO_6_Si_5_
13	16.08	D-Arabinose, tetrakis(trimethylsilyl) ether, thyroxine (isomer 1)	C_19_H_47_NO_5_Si_4_
14	16.22	D-Glucopyranose, 5TMS derivative	C_21_H_52_O_6_Si_5_
15	16.25	d-Galactose, 2,3,4,5,6-pentakis-O-(trimethylsilyl)-, o-methyloxyme, (1*Z*)-	C_22_H_55_NO_6_Si_5_
16	16.31	1,5-Anhydroglucitol, 4TMS derivative	C_18_H_44_O_5_Si_4_
17	16.39	D-Lyxose, 4TMS derivative	C_17_H_42_O_5_Si_4_
18	16.43	Ribitol, 5TMS derivative	C_20_H_52_O_5_Si_5_
19	16.52	meso-Erythritol, 4TMS derivative	C_16_H_42_O_4_Si_4_
20	16.55	D-Lyxose, 4TMS derivative	C_17_H_42_O_5_Si_4_
21	16.60	D-Mannitol, 6TMS derivative	C_24_H_62_O_6_Si_6_
22	16.64	D-Arabinose, tetrakis(trimethylsilyl) ether, ethyloxime (isomer 1)	C_19_H_47_NO_5_Si_4_
23	16.80	D-Lyxose, 4TMS derivative	C_17_H_42_O_5_Si_4_
24	16.88	Methyl.α.-D-ribofuranoside, 3TMS derivative	C_15_H_36_O_5_Si_3_
25	17.05	D-Glucopyranose, 5TMS derivative	C_21_H_52_O_6_Si_5_

*TMS: Trimethylsilyl group, ***t***_**R**_: retention time

For example, apples’ sweet flavour is largely due to D-fructose, D-galactose, and D-lyxose. Particularly, fructose, which is the predominant sugar in apples and sweeter than sucrose or glucose, has a significant impact on consumer choice (Wu et al. 2019). The importance of fructose derivatives to apple sweetness and overall palatability is further supported by the large relative levels of D-(-)-fructofuranose and D-fructose-O-methyl oxime.

For post-harvest storage, meso-erythritol, D-mannitol, and 1,5-anhydroglucitol can function as biochemical indicators of ripeness, senescence, and stress response. As osmoprotectants and antioxidants, for instance, mannitol and erythritol build up in reaction to oxidative or environmental stress (Ejaz et al. 2019, 2020; Anjum et al. 2023). Higher levels in samples that have been preserved could be a sign of stress signalling or tissue adaptation.

Small amounts of certain identified sugars, such as ribitol, D-psicofuranose, and D-arabinose-O-methyl oxime, may be involved in derivatization routes or cellular metabolism. In medical diagnostics, for example, 1,5-anhydroglucitol has been studied as a glycemic control marker, and ribitol is implicated in the pentose phosphate pathway and bacterial cell wall biosynthesis (Fiskesund et al. 2010; Cataldi and Lu 2025).

The variance in sugar concentration, especially the high sugar area in T4, points to possible variations in storage conditions, harvest ripeness, or variety characteristics. The apples’ chemical integrity, sweetness profile, and suitability for various processing or nutritional uses may all be impacted by these variations.

#### Antimicrobial activity

Antimicrobial activity test including some of the popular food born microorganisms namely *E. coli*, *S. aureus*, *C. albicans*, and *A. niger*, was tested against the formulated bioactive nanopackaging film with three different concentrations of orange peel extract and nanochitosan as a blank. Table 5 illustrates the antimicrobial activity, which reflected a low antimicrobial activity for nanochitsan against filamentous fungi (Hassan et al. 2022) and moderate antimicrobial activity against bacteria (Mirhosseini et al. 2023) with both types and unicellular fungi (Saied et al. 2023). In addition, adding the orange peel waste extract enhanced the antimicrobial activity of bioactive nanopackaging films in parallel with the increase in orange peel waste extract concentration. Indeed, the bioactive nanopackaging film containing 5% orange peel waste extract showed broad-spectrum antibacterial activity that was more than that of streptomycin (reference antibiotic) (Shehata et al. 2021; Selahvarzi et al. 2021). In addition, the antifungal activity was assigned against unicellular fungi for the film mentioned above, and against *A. niger*, the antimicrobial activity was recorded as low value that was also less than the reference antifungal drug (griseofulvin) (Hernández et al. 2021). Herein, the nanochitosan presented low antimicrobial activity that needs to be supported with orange peelwaste extract, which enhances the antimicrobial activity. In this context, the antimicrobial activity was recorded for all films against the bacterial strains; otherwise, for fungal strains, the unicellular fungi were more affected than the filamentous fungi.

**Table 5:** Antimicrobial activity *via* turbidimetric assay

Treatments	E. coli	S. aureus	C. albicans	A. niger
T2	45 ± 2*	36 ± 2	21 ± 2	5 ± 1
T3	51±	48 ± 2	38 ± 3	11 ± 1
T4	62 ± 3	61 ± 3	52 ± 4	16 ± 2
T5	79 ± 3	77 ± 4	67 ± 4	27 ± 2
Streptomycin (Antibacterial)	72 ± 3	61 ± 2	NA**	NA
Griseofulvin (antifungal)	NA	NA	66 ± 2	49 ± 2

*Antimicrobial activity %, **This antibiotic does not apply to this strain

#### Antioxidant activity

**Fig. 10:**
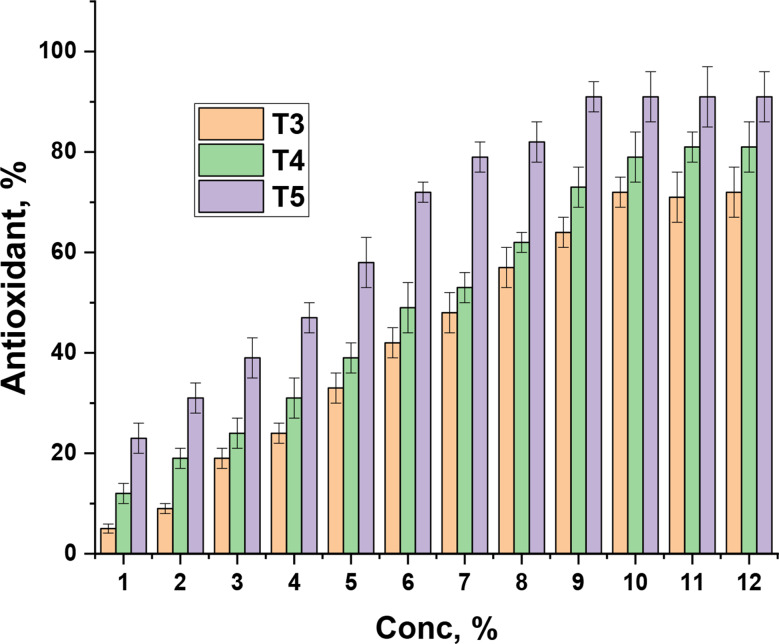
Antioxidant activity of nanopackaging films

The antioxidant activity of the prepared bioactive nanopackaging films comes from nanochitosan (Taşkın et al. 2021) and orange peel waste extract (Shehata et al. 2021). Indeed, the percentage of orange peel waste extract enhanced the film’s antioxidant behaviors, as shown in Fig. 10. The bioactive nanopackaging films exhibit antioxidant activity with concentrations of 7% that scavenge 50% of DPPH for bioactive nanopackaging films containing 1% orange peel waste extract. A film containing 3% reflects 53%, and the film containing 5% recorded 79%. Moreover, the high concentrations of bioactive nanopackaging films individually respond 72, 81, and 91% for bioactive nanopackaging films **T3**, **T4**, and **T5** extract, respectively. These observations emphasized that the orange peel waste extract induced the antioxidant activity that energetically goes with a nanochitosan (Fig. 10).

## Conclusion

Bioactive packaging films are widely used in safe envelopes, such as food. The edibility of these films is required to be safe for food and consumers. This work used the orange peel waste as a precious material for the nanocellulose and orange peel waste extract. In addition, the shrimp waste was used to produce nanochitosan. The formulation of the abovementioned ingredients has produced multifunctionality, such as antimicrobial and antioxidant activity. Moreover, the physicochemical and morphological aspects of the formulated films and neatly prepared ingredients, including nanochitosan and nanocellulose, were evaluated, and we observed significant changes in the physicochemical and morphological appearance. In addition, citrus polyphenols, such as phenolics and flavanones, are recognized as essential ingredients of packaging films to protect the fruits from deteriorationduring storage. Post-harvest losses are losses that occur between the harvest and the consumption stage. These losses can be physical, aesthetic, and nutritional, and become economic losses in the production chain. The results revealed that all treatments outperformed the control, especially treatments T4 and T5, concerning fruit decay percentage, weight loss, total acidity, fruit firmness, TSS, total sugars, and total anthocyanins.

## Electronic supplementary material

Below is the link to the electronic supplementary material.

Supplementary Material 1

## Data Availability

Data for this article, including characterization of materials and applications, are available from the corresponding author.
