# Supplementary material for: Bioactive preservative nano-packaging films based on food wastes of orange peels and Shrimp for apple (Malus domestica var. Anna) fruit quality and storage
Source: Bioresour Bioprocess. 2025 Jun 6;12(1):54. doi: 10.1186/s40643-025-00890-9 (PMC12144032; doi:10.1186/s40643-025-00890-9)
Supplement: Supplementary file 1 — Supplementary Material 1 [file 40643_2025_890_MOESM1_ESM.docx]

**Bioactive preservative nano-packaging films based on food wastes of orange peels and Shrimp for apple (*Malus domestica* var. Anna) fruit quality and storage**

**Mohamed S. Hasanin^1*^, Mahmoud Emam^2^, M.A. Ahmed^3^, F.M. Rohim^3^, M.A.A. Mohamed^4^, Housni El Saied^1^, Hamdy A. Z. Hussein^5^, A. Abdelkhalek^3*^**

^1^Cellulose& Paper Department, National Research Centre, El-Buhouth St., Dokki, 12622, Egypt

^2^Phytochemistry and Plant Systematics Department, National Research Centre, Dokki, Cairo, 12622, Egypt

^3^Horticultural Crops Technology Department, National Research Centre, Cairo, 12622, Egypt

^4^Fruit Handling Department, Horticulture Research Institute, Agricultural Research Center, Giza 12619, Egypt

^5^Horticulture Department, Faculty of Agriculture, Fayoum University, Fayoum 63514, Egypt

Corresponding author: Mohamed S. Hasanin, Email: [sido_sci@yahoo.com](mailto:sido_sci@yahoo.com)

A. Abdelkhalek, Email: [d.ammora@gmail.com](mailto:d.ammora@gmail.com)

**Supplementary**

**Table S-1: Total** **acidity (%), Total soluble solids (TSS) and TSS/Acidity over different storage periods of "Anna" cv. apple fruits influenced by some packaging materials**

| **Treatments (T)** | **Periods (P; Days)** | | | | | | | | | | **Mean (T)** |
| --- | --- | --- | --- | --- | --- | --- | --- | --- | --- | --- | --- |
|  | **0** | **15** | **30** | **45** | **60** | **75** | **90** | **105** | | **120** |  |
|  | **Total acidity (%)** | | | | | | | | | |  |
| **T1** | 0.71±0.015**^a^** | 0.70±0.006**^a^** | 0.68±0.015**^a^** | 0.69±0.006**^a^** | 0.66±0.015**^a^** | 0.66±0.012**^a^** | 0.62±0.015**^a^** | 0.63±0.015**^a^** | | 0.64±0.012**^a^** | **0.67**±**0.033^B^** |
| **T2** | 0.80±0.045**^a^** | 0.79±0.036**^a^** | 0.77±0.046**^a^** | 0.76±0.045**^a^** | 0.74±0.035**^a^** | 0.71±0.021**^a^** | 0.70±0.015**^a^** | 0.71±0.012**^a^** | | 0.71±0.012**^a^** | **0.74**±0.037**^A^** |
| **T3** | 0.64±0.036**^a^** | 0.63±0.029**^a^** | 0.61±0.021**^a^** | 0.61±0.017**^a^** | 0.60±0.015**^a^** | 0.58±0.010**^a^** | 0.56±0.015**^a^** | 0.58±0.020**^a^** | | 0.60±0.025**^a^** | **0.60**±0.025**^C^** |
| **T4** | 0.65±0.061**^a^** | 0.63±0.058**^a^** | 0.62±0.055**^a^** | 0.61±0.052**^a^** | 0.59±0.053**^a^** | 0.57±0.049**^a^** | 0.56±0.055**^a^** | 0.57±0.052**^a^** | | 0.60±0.047**^a^** | **0.60**±0.030**^C^** |
| **T5** | 0.72±0.026**^a^** | 0.73±0.025**^a^** | 0.71±0.029**^a^** | 0.70±0.031**^a^** | 0.68±0.021**^a^** | 0.65±0.026**^a^** | 0.63±0.021**^a^** | 0.63±0.017**^a^** | | 0.66±0.021**^a^** | **0.68**±0.039**^B^** |
| **Mean (P)** | **0.70**±**0.063^A^** | **0.70**±**0.066^A^** | **0.68**±**0.066^B^** | **0.67**±**0.063^B^** | **0.65**±**0.061^C^** | **0.63**±0.058**^D^** | **0.61**±**0.057^F^** | **0.62**±0.054**^E^** | | **0.64**±**0.048^D^** |  |
| Significance | P= ** | | | T*P = NS | | | | | | | **T= **** |
| **Total soluble solids (TSS) content** | | | | | | | | | | | |
| **T1** | 8.77±0.15p | 8.90±0.10op | 9.10±0.17m-o | 9.20±0.1m-o | 9.23±.06lm | 9.40±0.1lm | 9.23±0.06mn | 9.17±0.06m-o | | 9.00±0.0n-p | **9.11**±**0.18E** |
| **T2** | 11.13±0.15e | 11.33±0.15de | 11.60±0.20cd | 11.70±0.2bc | 11.83±0.21a-c | 12.03±0.21a | 11.93±0.12ab | 11.80±0.1a-c | | 11.67±0.12bc | **11.67**±**0.27A** |
| **T3** | 9.60±0.52l | 10.17±0.12i-k | 10.30±0.1h-j | 10.30±0.1h-l | 10.53±0.49f-h | 11.10±0.10e | 10.80±0.2f | 10.67±0.12fg | | 10.47±0.12g-i | **10.44**±**0.4C** |
| **T4** | 9.53l±0.06 | 9.93±0.12mn | 10.11±0.1jk | 10.29±0.19h-j | 10.30±0.1h-j | 10.43±0.15g-i | 10.17±0.06i-k | 10.00±0.0jk | | 10.57±0.49f-h | **10.15**±**0.29D** |
| **T5** | 10.80±0.20f | 11.10±0.10e | 11.30±0.1de | 11.33±0.15de | 11.57±0.06cd | 11.73±0.12a-c | 11.53±0.12cd | 11.33±0.12de | | 11.17±0.15e | **11.32**±**0.26B** |
| **Mean (P)** | **9.97**±**0.98F** | **10.29**±0.98**E** | **10.48**±1.00**D** | **10.57**±**0.99CD** | **10.69**±1.05**BC** | **10.94**±**1.06A** | **10.73**±**1.08B** | **10.59**±**1.05CD** | | **10.57**±**1.00CD** |  |
| Significance | P= ** | | | T*P = ** | | | | | | | **T= **** |
| **TSS/Acidity** | | | | | | | | | | | |
| **T1** | 12.29±0.25y | 12.65±0.07y | 13.32±0.36x | 13.40±0.08wx | 14.07±0.41u | 14.32±0.14tu | 14.98±0.47p-s | 14.48±0.35r-u | 13.99±0.25u-w | | **13.72**±**0.83B** |
| **T2** | 14.00±0.62u-w | 14.36±0.61s-u | 15.09±0.66p-r | 15.49±0.67n-p | 16.08±0.50k-n | 16.87±0.27ij | 17.13±0.23f-i | 16.70±0.17i-k | 16.36±0.23k-n | | **15.79**±**1.06A** |
| **T3** | 15.00±0.23p-s | 16.08±0.94k-n | 16.80±0.42ij | 16.90±0.64ij | 17.67±1.27d-g | 19.14±0.45a | 19.19±0.86a | 18.41±0.82b | 17.57±0.94e-h | | **17.42**±**1.31A** |
| **T4** | 14.74±1.23q-t | 15.78±1.54m-o | 16.48±1.55i-l | 16.97±1.65h-j | 17.56±1.67e-h | 18.30±1.72b-d | 18.38±1.83bc | 17.64±1.53e-g | 17.76±1.09c-f | | **17.07**±**1.14A** |
| **T5** | 15.01±0.39p-s | 15.29±0.6o-q | 15.86±0.67l-o | 16.13±0.64k-n | 17.10±0.46f-i | 18.07±0.62b-e | 18.41±0.49b | 18.00±0.43b-e | 17.02±0.60g-j | | **16.77**±**1.18A** |
| **Mean (P)** | **14.21**±**1.15H** | **14.83**±1.38**G** | **15.51**±1.39**F** | **15.78**±1.46**E** | **16.50**±1.50**D** | **17.34**±1.87**B** | **17.62**±1.65**A** | **17.04**±1.57**C** | **16.54**±1.52**D** | |  |
| Significance | P= ** | | | T*P = * | | | | | | | **T= **** |

T1= Control (dipping in water), T2=1% Nano Chitosan, T3= 1% Nano Chitosan + 1% Nano Cellulose + 1% Orange Peel Waste extract, T4= 1% Nano Chitosan + 1% Nano Cellulose + 3% Orange Peel Waste extract, T5= 1% Nano Chitosan + 1% Nano Cellulose + 5% Orange Peel Waste extract.

Mean values with different letters in each column are significant (at *p ≤* 0.05); ** = Significant at the 1% level of probability. Lowercase letters in the same column compare the average values of treatments (for the same storage period). Uppercase letters in a row Mean (P) represent a comparison of mean values between different periods (for all treatments). Uppercase letters in the last column represent a comparison of the mean values between different treatments (for all storage periods).

**Table S-2: Decay (%) and Weight Loss (%) over different storage periods of "Anna" cv. apple fruits influenced by some packaging materials**

| **Treatments (T)** | **Periods (P; Days)** | | | | | | | | | **Mean (T)** |
| --- | --- | --- | --- | --- | --- | --- | --- | --- | --- | --- |
|  | **0** | **15** | **30** | **45** | **60** | **75** | **90** | **105** | **120** |  |
|  | **Decay (%)** | | | | | | | | |  |
| **T1** | 0.00±0.0n | 0.00±0.0n | 11.31±2.93j | 13.20±3.08ij | 15.28±1.98hi | 15.55±1.09hi | 25.92±3.26de | 28.49±4.68cd | 40.53±0.53a | **16.70**±**12.45A** |
| **T2** | 0.00±0.0n | 0.00±0.0n | 0.00±0.0n | 3.32±0.45m | 7.03±0.51l | 10.67±0.78jk | 18.67±1.49fg | 25.09±4.68e | 32.92±0.57b | **10.85**±**11.43C** |
| **T3** | 0.00±0.0n | 0.00±0.0n | 2.30±1.0mn | 8.41±0.58kl | 12.24±2.62j | 16.37±2.59gh | 26.55±1.78de | 31.21±3.99b | 32.70±0.52b | **14.42**±**12.34B** |
| **T4** | 0.00±0.0n | 0.00±0.0n | 0.00±0.0n | 3.74±1.27m | 8.60±0.75kl | 15.44±1.24hi | 19.77±0.93f | 26.56±1.53de | 31.37±1.62b | **11.72**±**11.4C** |
| **T5** | 0.00±0.0n | 0.00±0.0n | 0.00±0.0n | 7.55±0.7l | 11.42±2.12j | 15.39±1.32hi | 20.83±2.60f | 25.58±2.27e | 30.16±0.35bc | **12.33**±**10.83C** |
| **Mean (P)** | **0.00**±**0.0H** | **0.00**±0.0**H** | **2.72**±**4.9G** | **7.25**±4.02**F** | **10.91**±3.22**E** | **14.68**±**2.28D** | **22.35**±**3.64C** | **27.38**±2.5**B** | **33.54**±4.07**A** |  |
| Significance | P= ** | | | T*P = ** | | | | | | **T= **** |
| **Weight Loss (%)** | | | | | | | | | | |
| **T1** | 0.00±0.0t | 1.87±0.29qr | 2.54±0.33n-p | 3.04±0.09l | 3.84±0.13k | 4.63±0.19j | 5.82±0.36h | 7.05±0.54b-d | 8.35±0.17a | **4.13**±**2.48A** |
| **T2** | 0.00±0.0t | 1.51±0.13rs | 2.28±0.26op | 2.86±0.08l-n | 3.59±0.12k | 4.29±0.15j | 5.51±0.25hi | 6.38±0.09fg | 6.75±0.29d-f | **3.68**±**2.14B** |
| **T3** | 0.00±0.0t | 1.54±0.11rs | 2.34±0.15op | 2.75±0.48l-n | 3.45±0.44k | 4.29±0.22j | 5.45±0.3hi | 6.21±0.03g | 6.87±0.42c-e | **3.66**±**2.13B** |
| **T4** | 0.00±0.0t | 1.42±0.08s | 2.18±0.05pq | 2.94±0.03lm | 3.76±0.03k | 4.48±0.03j | 5.69±0.05hi | 6.53±0.25e-g | 7.29±0.12b | **3.81**±**2.29B** |
| **T5** | 0.00±0.0t | 1.67±0.9rs | 2.36±0.13op | 2.62±0.54m-o | 3.76±0.19k | 4.43±0.22j | 5.31±0.27i | 6.62±0.27ef | 7.15±0.02bc | **3.77**±**2.22B** |
| **Mean (P)** | **0.00**±0.0**I** | **1.60**±0.18**H** | **2.34**±**0.13G** | **2.84**±**0.17F** | **3.68**±0.16**E** | **4.42**±**0.14D** | **5.56**±**0.2C** | **6.56**±0.32**B** | **7.28**±**0.63A** |  |
| Significance | P= ** | | | T*P = ** | | | | | | **T= **** |

T1= Control (dipping in water), T2=1% Nano Chitosan, T3= 1% Nano Chitosan + 1% Nano Cellulose + 1% Orange Peel Waste extract, T4= 1% Nano Chitosan + 1% Nano Cellulose + 3% Orange Peel Waste extract, T5= 1% Nano Chitosan + 1% Nano Cellulose + 5% Orange Peel Waste extract

Mean values with different letters in each column are significant (at *p ≤* 0.05); ** = Significant at the 1% level of probability. Lowercase letters in the same column compare the average values of treatments (for the same storage period). Uppercase letters in a row Mean (P) represent a comparison of mean values between different periods (for all treatments). Uppercase letters in the last column represent a comparison of the mean values between different treatments (for all storage periods).

**Table S-3: Total chlorophyll content (mg g^-1^ FW) and Anthocyanin content over different storage periods of "Anna" cv. apple fruits influenced by some packaging materials**

| **Treatments (T)** | **Periods (P; Days)** | | | | | | | | | **Mean (T)** |
| --- | --- | --- | --- | --- | --- | --- | --- | --- | --- | --- |
|  | **0** | **15** | **30** | **45** | **60** | **75** | **90** | **105** | **120** |  |
|  | **Total chlorophyll content (mg g^-1^ FW)** | | | | | | | | |  |
| **T1** | 2.66±0.0a | 2.54±0.09ab | 2.48±0.11b-d | 2.42±0.12b-e | 2.34±0.12d-g | 2.27±0.15e-i | 2.15±0.21i-l | 2.07±0.25j-o | 2.03±0.23k-p | **2.33**±**0.2A** |
| **T2** | 2.66±0.0a | 2.56±0.14ab | 2.50±0.12ab | 2.45±0.12b-d | 2.41±0.09b-e | 2.37±0.08c-f | 2.32±0.06d-h | 2.12±0.28i-m | 2.24±0.04f-j | **2.40**±**0.16A** |
| **T3** | 2.66±0.0a | 2.27±0.12e-i | 2.17±0.14h-k | 2.10±0.15j-n | 2.05±0.13k-p | 1.99±0.10l-p | 1.94±0.09 | 1.91±0.08o-t | 1.81±0.07r-u | **2.10**±**0.24B** |
| **T4** | 2.66±0.0a | 2.18±0.15g-k | 2.14±0.16i-l | 2.05±0.16k-p | 1.99±0.16l-p | 1.95±0.14m-r | 1.88±0.14 | 1.92±0.07o-s | 1.77±0.07s-w | **2.06**±**0.24B** |
| **T5** | 2.66±0.0a | 1.93±0.15o-s | 1.88±0.13 | 1.82±0.08r-u | 1.75±0.7t-w | 1.69±0.07u-x | 1.62±0.04u-x | 1.54±0.03xy | 1.48±0.03y | **1.82**±**0.33C** |
| **Mean (P)** | **2.66**±**0.0A** | **2.30**±0.26**B** | **2.23**±0.26**B** | **2.17**±**0.26C** | **2.11**±0.27**CD** | **2.06**±**0.27D** | **1.98**±**0.27E** | **1.91**±0.23**F** | **1.87**±0.29**F** |  |
| Significance | P= ** | | | T*P = ** | | | | | | **T= **** |
| **Total Anthocyanin content** | | | | | | | | | | |
| **T1** | 0.14±0.01z | 0.14±0.0z | 0.15±0.01yz | 0.15±0.01yz | 0.16±0.0u-y | 0.17±0.01t-w | 0.17±0.0t-w | 0.18±0.01s-u | 0.18±0.01s-u | **0.16**±0.01**C** |
| **T2** | 0.14±0.01z | 0.16±0.01u-y | 0.16±0.01u-y | 0.17±0.01t-w | 0.17±0.01t-w | 0.19±0.01n-p | 0.19±0.01n-p | 0.19±0.0n-p | 0.20±0.01mn | **0.17**±**0.02C** |
| **T3** | 0.14±0.01z | 0.19±0.01n-p | 0.20±0.01mn | 0.20±0.01mn | 0.22±0.0jk | 0.23±0.0jk | 0.24±0.01g-i | 0.24±0.01g-i | 0.25±0.01e-g | **0.21**±**0.03B** |
| **T4** | 0.14±0.01z | 0.20±0.01mn | 0.20±0.02mn | 0.20±0.01mn | 0.21±0.02lm | 0.22±0.01kl | 0.22±0.01kl | 0.24±0.01g-i | 0.26±0.01ef | **0.21**±**0.03B** |
| **T5** | 0.14±0.01z | 0.24±0.04g-i | 0.25±0.03f-h | 0.25±0.03f-h | 0.26±0.03de | 0.27±0.03cd | 0.28±0.03bc | 0.29±0.03b | 0.30±0.02a | **0.25**±**0.05A** |
| **Mean (P)** | **0.14**±0.0**I** | **0.19**±**0.04H** | **0.19**±**0.04G** | **0.20**±**0.04F** | **0.20**±**0.04E** | **0.21**±**0.04D** | **0.22**±**0.04C** | **0.23**±**0.04B** | **0.24**±**0.05A** |  |
| Significance | P= ** | | | T*P = ** | | | | | | **T= **** |

T1= Control (dipping in water), T2=1% Nano Chitosan, T3= 1% Nano Chitosan + 1% Nano Cellulose + 1% Orange Peel Waste extract, T4= 1% Nano Chitosan + 1% Nano Cellulose + 3% Orange Peel Waste extract, T5= 1% Nano Chitosan + 1% Nano Cellulose + 5% Orange Peel Waste extract

Mean values with different letters in each column are significant (at *p ≤* 0.05); ** = Significant at the 1% level of probability. Lowercase letters in the same column compare the average values of treatments (for the same storage period). Uppercase letters in a row Mean (P) represent a comparison of mean values between different periods (for all treatments). Uppercase letters in the last column represent a comparison of the mean values between different treatments (for all storage periods).

**Table S-4: Total sugars (%) and Vitamin C content (mg /100 ml juice) over different storage periods of "Anna" cv. apple fruits influenced by some packaging materials**

| **Treatments (T)** | **Periods (P; Days)** | | | | | | | | | **Mean (T)** |
| --- | --- | --- | --- | --- | --- | --- | --- | --- | --- | --- |
|  | **0** | **15** | **30** | **45** | **60** | **75** | **90** | **105** | **120** |  |
|  | **Total sugars (%)** | | | | | | | | |  |
| **T1** | 7.53±0.17n-p | 7.68±0.03j-o | 7.72±0.03j-o | 7.77±0.05h-o | 7.78±0.06h-o | 7.76±0.04h-o | 7.60±0.4l-p | 7.55±0.06m-p | 7.45±0.10o-q | **7.65**±**0.11A** |
| **T2** | 7.71±0.13j-o | 7.63±0.16k-p | 7.58±0.19l-p | 7.83±0.13g-n | 7.84±0.26f-n | 7.90±0.29e-m | 7.72±0.29j-o | 7.31±0.44pq | 7.18±0.49q | **7.63**±**0.23A** |
| **T3** | 7.63±0.0k-p | 7.68±0.13j-o | 7.87±0.31f-n | 7.98±0.47c-k | 8.09±0.55b-i | 8.12±0.70b-h | 8.01±0.67c-j | 7.72±0.62j-o | 7.86±0.53f-n | **7.88**±**0.17A** |
| **T4** | 7.63±0.0k-p | 7.74±0.04i-o | 7.83±0.08g-n | 8.00±0.13c-j | 8.17±0.21b-g | 8.24±0.28a-e | 8.29±0.31a-c | 7.99±0.19c-k | 7.93±0.17d-l | **7.98**±**0.21A** |
| **T5** | 7.63±0.0k-p | 7.87±0.12f-n | 8.11±0.13b-h | 8.31±0.16a-c | 8.53±0.12a | 8.55±0.13a | 8.44±0.13ab | 8.27±0.24a-d | 8.19±0.27b-f | **8.21**±**0.29A** |
| **Mean (P)** | **7.63**±**0.06C** | **7.72**±**0.09BC** | **7.82**±**0.19B** | **7.98**±**0.21A** | **8.08**±**0.3A** | **8.11**±**0.31A** | **8.01**±**0.36A** | **7.77**±**0.37BC** | **7.72**±**0.4BC** |  |
| Significance | P= ** | | | T*P = ** | | | | | | **T= NS** |
| **Vitamin C (mg /100 ml juice)** | | | | | | | | | | |
| **T1** | 0.032±0.00a | 0.034±0.0a | 0.033±0.01a | 0.037±0.0a | 0.033±0.01a | 0.032±0.01a | 0.032±0.0a | 0.034±0.01a | 0.036±0.0a | **0.034**±**0.0A** |
| **T2** | 0.037±0.01a | 0.038±0.01a | 0.035±0.01a | 0.034±0.01a | 0.034±0.01a | 0.032±0.01a | 0.031±0.0a | 0.030±0.0a | 0.031±0.0a | **0.033**±**0.0A** |
| **T3** | 0.033±0.01a | 0.032±0.0a | 0.032±0.01a | 0.030±0.0a | 0.029±0.0a | 0.028±0.0a | 0.028±0.0a | 0.031±0.0a | 0.032±0.0a | **0.031**±**0.0A** |
| **T4** | 0.024±0.01a | 0.023±0.01a | 0.025±0.01a | 0.024±0.01a | 0.025±0.01a | 0.023±0.01a | 0.024±0.01a | 0.024±0.01a | 0.025±0.01a | **0.024**±**0.0B** |
| **T5** | 0.029±0.01a | 0.030±0.0a | 0.028±0.01a | 0.028±0.01a | 0.026±0.01a | 0.028±0.0a | 0.027±0.0a | 0.029±0.0a | 0.030±0.0a | **0.028**±**0.0AB** |
| Mean (P) | 0.031±0.01A | 0.031±0.01A | 0.031±0.004A | 0.031±0.01A | 0.029±0.004A | 0.029±0.004A | 0.028±0.003A | 0.030±0.004A | 0.031±0.004A |  |
| Significance | P= NS | | | T*P = NS | | | | | | **T= **** |

T1= Control (dipping in water), T2=1% Nano Chitosan, T3= 1% Nano Chitosan + 1% Nano Cellulose + 1% Orange Peel Waste extract, T4= 1% Nano Chitosan + 1% Nano Cellulose + 3% Orange Peel Waste extract, T5= 1% Nano Chitosan + 1% Nano Cellulose + 5% Orange Peel Waste extract

Mean values with different letters in each column are significant (at *p ≤* 0.05); ** = Significant at the 1% level of probability. Lowercase letters in the same column compare the average values of treatments (for the same storage period). Uppercase letters in a row Mean (P) represent a comparison of mean values between different periods (for all treatments). Uppercase letters in the last column represent a comparison of the mean values between different treatments (for all storage periods).

**Table S-5: Fruit firmness and Total pectin content over different storage periods of "Anna" cv. apple fruits influenced by some packaging materials**

| **Treatments (T)** | **Periods (P; Days)** | | | | | | | | | **Mean (T)** |
| --- | --- | --- | --- | --- | --- | --- | --- | --- | --- | --- |
|  | **0** | **15** | **30** | **45** | **60** | **75** | **90** | **105** | **120** |  |
|  | **Fruit firmness** | | | | | | | | |  |
| **T1** | 14.49±0.58ab | 13.51±0.11e-g | 13.07±0.12h-j | 12.50±0.42k-m | 12.11±0.65mn | 11.35±0.51q | 10.01±0.97^st^ | 9.08±0.49u | 8.78±0.37u | **11.66**±1.89**D** |
| **T2** | 14.85±0.06a | 13.50±0.08e-g | 13.12±0.05g-j | 12.56±0.38kl | 12.18±0.31l-n | 11.59±0.12o-q | 10.97±0.32r | 10.13±0.09s | 9.66±0.14u | **12.06**±**1.57C** |
| **T3** | 14.75±0.12a | 13.66±0.28d-f | 13.16±0.06g-i | 12.80±0.29i-k | 12.55±0.24kl | 12.11±0.27mn | 11.51±0.32pq | 10.34±0.10rs | 10.09±0.13s | **12.33**±**1.43C** |
| **T4** | 14.72±0.17a | 14.04±0.14c | 13.79±0.21c-e | 13.31±0.39f-h | 12.96±0.39h-j | 12.43±0.51k-m | 11.85±0.47n-p | 11.42±0.51q | 10.97±0.62r | **12.83**±**1.19B** |
| **T5** | 14.82±0.0a | 14.46±0.21ab | 14.15±0.04bc | 13.97±0.15cd | 13.61±0.14d-f | 13.20±0.22gh | 12.77±0.25jk | 12.45±0.15k-m | 11.92±0.08no | **13.48**±**0.91A** |
| **Mean (P)** | **14.73**±**0.14A** | **13.83**±**0.41B** | **13.46**±**0.49C** | **13.03**±0.62**D** | **12.68**±**0.62E** | **12.14**±**0.73F** | **11.42**±**1.03G** | **10.69**±**1.29H** | **10.29**±1.21**I** |  |
| Significance | P= ** | | | T*P = ** | | | | | | **T= **** |
| **Total pectin content** | | | | | | | | | | |
| **T1** | 0.22±0.0y | 0.38±0.02o-q | 0.43±0.02l-n | 0.50±0.01ij | 0.56±0.03gh | 0.61±0.04ef | 0.66±0.03cd | 0.73±0.02b | 0.80±0.04a | **0.54**±**0.17A** |
| **T2** | 0.22±0.0y | 0.30±0.02s-u | 0.35±0.02qr | 0.43±0.05l-n | 0.47±0.03j-l | 0.53±0.04hi | 0.61±0.04ef | 0.64±0.04de | 0.68±0.03c | **0.47**±**0.15B** |
| **T3** | 0.22±0.0y | 0.25±0.03w-y | 0.27±0.01u-x | 0.30±0.02s-u | 0.34±0.04q-s | 0.36±0.04qr | 0.40±0.01n-p | 0.42±0.03mn | 0.46±0.01k-m | **0.34**±**0.08D** |
| **T4** | 0.22±0.0y | 0.27±0.02u-x | 0.30±0.01s-u | 0.34±0.02q-s | 0.38±0.02o-q | 0.42±0.0mn | 0.46±0.01k-m | 0.50±0.02ij | 0.58±0.03fg | **0.38**±**0.11C** |
| **T5** | 0.22±0.0y | 0.24±0.03xy | 0.29±0.05u-x | 0.33±0.04r-t | 0.37±0.02pq | 0.41±0.01no | 0.47±0.01j-l | 0.52±0.05hi | 0.61±0.01ef | **0.38**±**0.12C** |
| **Mean (P)** | **0.22**±0.0**I** | **0.29**±**0.06H** | **0.33**±**0.07G** | **0.38**±**0.08F** | **0.42**±0.09**E** | **0.47**±**0.10D** | **0.52**±**0.11C** | **0.56**±**0.12B** | **0.63**±0.13**A** |  |
| Significance | P= ** | | | T*P = ** | | | | | | **T= **** |

T1= Control (dipping in water), T2=1% Nano Chitosan, T3= 1% Nano Chitosan + 1% Nano Cellulose + 1% Orange Peel Waste extract, T4= 1% Nano Chitosan + 1% Nano Cellulose + 3% Orange Peel Waste extract, T5= 1% Nano Chitosan + 1% Nano Cellulose + 5% Orange Peel Waste extract

Mean values with different letters in each column are significant (at *p ≤* 0.05); ** = Significant at the 1% level of probability. Lowercase letters in the same column compare the average values of treatments (for the same storage period). Uppercase letters in a row Mean (P) represent a comparison of mean values between different periods (for all treatments). Uppercase letters in the last column represent a comparison of the mean values between different treatments (for all storage periods).
